# MMP12 induces fibrogenic macrophage differentiation to drive granuloma-associated cardiac fibrosis

**DOI:** 10.1093/ajrccm/aamag354

**Published:** 2026-07-29

**Authors:** Mario Mazić, Clarice X Lim, Piyal Saha, Thomas El-Jammal, Roko Šango, Tayeba Ghazi, Armela Jahić, Paul Ettel, Joanna Jager, Angeliki Asimaki, Mary N Sheppard, Hannes Todt, Dagmar Kratky, Markus Hengstschläger, Wenjin Yang, Thomas Weichhart

**Affiliations:** Center for Pathobiochemistry and Genetics, Medical University of Vienna, Vienna, Austria; Center for Pathobiochemistry and Genetics, Medical University of Vienna, Vienna, Austria; Center for Pathobiochemistry and Genetics, Medical University of Vienna, Vienna, Austria; Center for Pathobiochemistry and Genetics, Medical University of Vienna, Vienna, Austria; Laboratory of Tissue Biology and Therapeutic Engineering, CNRS UMR5305, University Claude Bernard Lyon 1, Institute of Biology and Chemistry of Proteins, Lyon, France; Center for Pathobiochemistry and Genetics, Medical University of Vienna, Vienna, Austria; Centre for Microbiology and Environmental Systems Science, University of Vienna, Vienna, Austria; Center for Pathobiochemistry and Genetics, Medical University of Vienna, Vienna, Austria; Center for Pathobiochemistry and Genetics, Medical University of Vienna, Vienna, Austria; Center for Pathobiochemistry and Genetics, Medical University of Vienna, Vienna, Austria; Clinical Cardiology Academic Group, Molecular and Clinical Research Science, Institute St George’s University of London, London, United Kingdom; Clinical Cardiology Academic Group, Molecular and Clinical Research Science, Institute St George’s University of London, London, United Kingdom; Clinical Cardiology Academic Group, CRY Centre for Cardiac Pathology, St George’s University of London, London, United Kingdom; Center for Physiology and Pharmacology, Medical University of Vienna, Vienna, Austria; Gottfried Schatz Research Center, Molecular Biology and Biochemistry, Medical University of Graz, Graz, Austria; Center for Pathobiochemistry and Genetics, Medical University of Vienna, Vienna, Austria; Foresee Pharmaceuticals Co., Ltd., Research and Development, Taipei City, Taiwan; Center for Pathobiochemistry and Genetics, Medical University of Vienna, Vienna, Austria

**Keywords:** cardiac sarcoidosis, fibrosis, macrophages, MMP12

## Abstract

**Rationale:**

Cardiac sarcoidosis is a granulomatous inflammatory disease that frequently progresses to irreversible myocardial fibrosis and lethal conduction abnormalities. While mechanistic target of rapamycine complex 1 (mTORC1)–dependent macrophage activation has been implicated in this process, exactly how granulomatous inflammation is mechanistically converted into fibrotic remodeling at the level of macrophage effector function remains unknown.

**Methods:**

We employed a mouse model with myeloid-specific *Tsc2* deletion that recapitulates key features of human cardiac sarcoidosis. Single-cell RNA sequencing, bioinformatic analyses, histopathology, *in vitro* functional studies in murine and human macrophages, and *in vivo* pharmacologic inhibition were integrated to define macrophage-intrinsic mechanisms driving granuloma-associated cardiac fibrosis.

**Measurements and Main Results:**

Constitutive mTORC1 activation induced a fibrogenic macrophage (FibMac) population in the heart characterized by expression of *Cd63*, *Spp1*, *Gpnmb*, and *Fabp5* and arising through a TGF-β–dependent monocyte-to-FibMac differentiation process. We identified matrix metalloproteinase 12 (MMP12) as a macrophage-intrinsic inducer and dominant effector of this program. Recombinant MMP12 was sufficient to enforce fibrogenic macrophage differentiation and promote macrophage clustering and epithelioid features in both mouse and human macrophages. Conversely, selective MMP12 inhibition disrupted granuloma architecture, reduced fibroblast activation, and attenuated myocardial fibrosis *in vivo*, with concomitant improvement of cardiac conduction. In human cardiac sarcoidosis tissue, MMP12 colocalized with FibMac markers within granulomas, and reanalysis of published human fibrosis datasets revealed MMP12 expression within analogous fibrogenic macrophage populations.

**Conclusions:**

Our findings uncover MMP12 as a macrophage-intrinsic inducer and sustainer that links fibrogenic macrophage identity to granuloma architecture and cardiac fibrosis in sarcoidosis. Targeting this effector axis provides a therapeutic strategy distinct from upstream mTORC1 inhibition.

At a Glance Commentary **Current Scientific Knowledge on the Subject:** Cardiac sarcoidosis is a granulomatous inflammatory disease that frequently ­progresses to myocardial fibrosis and conduction abnormalities. Although mTORC1-dependent macrophage activation has been implicated in sarcoidosis pathogenesis, the macrophage-intrinsic mechanisms linking granuloma formation to fibrotic remodeling remain poorly understood.

**What This Study Adds to the Field:** We identify a fibrogenic macrophage population in mouse and human cardiac sarcoidosis ­characterized by expression of CD63, SPP1, GPNMB, and FABP5. We further identify MMP12 as a macrophage-intrinsic inducer and sustainer of this program that promotes granuloma organization and cardiac fibrosis, and show that selective MMP12 inhibition attenuates fibrosis and improves cardiac conduction *in vivo*.

## Introduction

The extracellular matrix (ECM) is a dynamic macromolecular network that provides structural integrity and biochemical signals to cells, tissues, and organs.^1^^,^^2^ In the heart, a coordinated ECM turnover maintains myocardial architecture and electrical and mechanical function.^3-5^ Continuous ECM remodeling by immune and mesenchymal cells is essential for tissue homeostasis and wound repair. However, persistent injury or chronic inflammation disrupts this balance and drives pathological remodeling, culminating in fibrosis. Cardiac fibrosis is characterized by excessive and disorganized ECM deposition,^6^^,^^7^ increased tissue stiffness, cardiomyocyte dysfunction, and impaired electrical conduction, ultimately leading to arrhythmias, ventricular dysfunction, and sudden cardiac death.^8-10^

Despite its major contribution to morbidity and mortality, the cellular and molecular mechanisms that drive cardiac fibrosis remain incompletely understood, and no durable antifibrotic therapies are currently available. Macrophages have emerged as central regulators of fibrotic remodeling across tissues. Recent work identified a fibrogenic macrophage population in experimental and human lung and liver fibrosis characterized by expression of *Cd9*, *Cd63*, *Spp1*, *Gpnmb*, and *Fabp5.*^11^ These macrophages cooperate with other immune cells to establish profibrotic cytokine milieus, including TGF-β, IL-17A, and GM-CSF. However, while upstream signals that promote fibrogenic macrophage differentiation are increasingly well defined, the macrophage-intrinsic effector mechanisms that physically remodel tissue architecture and sustain fibrosis remain largely unknown.

Cardiac sarcoidosis (CS) is a granulomatous inflammatory disease marked by immune cell infiltration, formation of noncaseating granulomas, and progressive myocardial fibrosis.^12^ Cardiac involvement is estimated to occur in up to 25% of patients with systemic sarcoidosis,^13^ yet often remains clinically silent until advanced stages, when patients present with conduction abnormalities, malignant arrhythmias, heart failure, or sudden cardiac death.^14^ The transition from granulomatous inflammation to fibrosis in CS is poorly understood, yet it is a major determinant of disease progression and clinical outcome. Although diverse genetic and environmental factors have been implicated in sarcoidosis pathogenesis,^15^ how granulomatous immune responses become mechanistically coupled to fibrotic remodeling in the heart remains unresolved.

We previously demonstrated that myeloid-specific deletion of *Tsc2* in mice causes chronic mechanistic target of rapamycine complex 1 (mTORC1) activation in macrophages and recapitulates key features of human CS, including granuloma formation, progressive fibrosis, and cardiac dysfunction.^16^ However, how this inflammatory state is converted into stable granuloma architecture and irreversible fibrotic remodeling has remained unclear. Here, we uncovered a mTORC1-driven differentiation process of monocytes giving rise to fibrogenic, granuloma-forming macrophages. We further identify matrix metalloproteinase 12 (MMP12) as a macrophage-intrinsic inducer that is able to initiate and maintain this population and is sufficient to promote macrophage clustering, epithelioid differentiation, and ECM remodeling. Pharmacologic inhibition of MMP12 disrupts granulomas, reduces myocardial fibrosis, and improves cardiac conduction *in vivo*, establishing MMP12 as a macrophage-intrinsic inducer and effector that couples fibrogenic macrophage differentiation to cardiac pathology in sarcoidosis.

## Methods

Methods are detailed in the online supplement and only briefly summarized here.

### Human participants

Formalin-fixed, paraffin-embedded tissue blocks from patients with CS and cardiac tissue from healthy donors were used to evaluate the presence of fibrogenic macrophages.

### Mouse model

Tsc2^fl/fl^CD11c^cre/+^ (denoted as cre/+) mice were used as previously described.^16^  *Mmp12* full-body knockout mice (MMP12^−^/^−^) were acquired from The Jackson Laboratory. The official Austrian institutional ethics board approved all animal experiments conducted in this study.

### Electrocardiography

Electrocardiographic (ECG) recordings were obtained from anesthetized mice using 6 subcutaneously placed electrodes in a standard limb lead configuration.

### Transcriptomics analysis

Processing and analysis of single-cell RNA sequencing (scRNA-seq) were conducted using the “Seurat” package in R software. A total of 6 samples (+/+ *n* = 3; cre/+ *n* = 3) were processed. Clustering of cells was done using the Louvain algorithm, and marker genes were identified with the Wilcoxon rank-sum test. False discovery rate–adjusted *P* values <.05 were considered statistically significant. Further downstream analysis included gene set enrichment analysis, protein–protein interaction network analysis, and pseudotime trajectory inference.

### *In vivo* experiments

Mice were randomly assigned groups, and no major sex specific differences were observed between groups. Additional details regarding experimental setup and *in vivo* treatment are available in the online supplement.

### Validation

Multicolor immunofluorescence stainings were performed to validate genes identified from bioinformatics analysis.

## Results

### Single-cell transcriptomics identifies a conserved fibrogenic macrophage population

We previously showed that deletion of *Tsc2* in CD11c-expressing cells (Tsc2^fl/fl^CD11c^cre/+^; denoted as cre/+ mice) promoted macrophage infiltration and spontaneous development of cardiac granulomas (Figure 1A, Figure S1A).^16^ The macrophages co-expressed pS6 (mTORC1 activity marker) and MAC2 (activated macrophage marker), revealing high mTORC1 activity in the infiltrating granulomatous macrophages (Figure S1B, C). Another prominent feature of this model is progressive nonresolving cardiac fibrosis (Figure 1B, Figure S1D). Interestingly, many infiltrating macrophages were located next to large Collagen 1 deposits (Figure 1C). Additionally, we noted intense scarring and thickening of the epicardial layer (Figure S1E, F) in the heart of the cre/+ mice. The numbers of cardiac myofibroblasts were highly increased, as seen by the increased number of fibroblast activation protein alpha (FAPα)– and alpha-smooth muscle actin (αSMA)–positive cells in all cardiac regions (Figure S1G-J).

**Figure 1:**
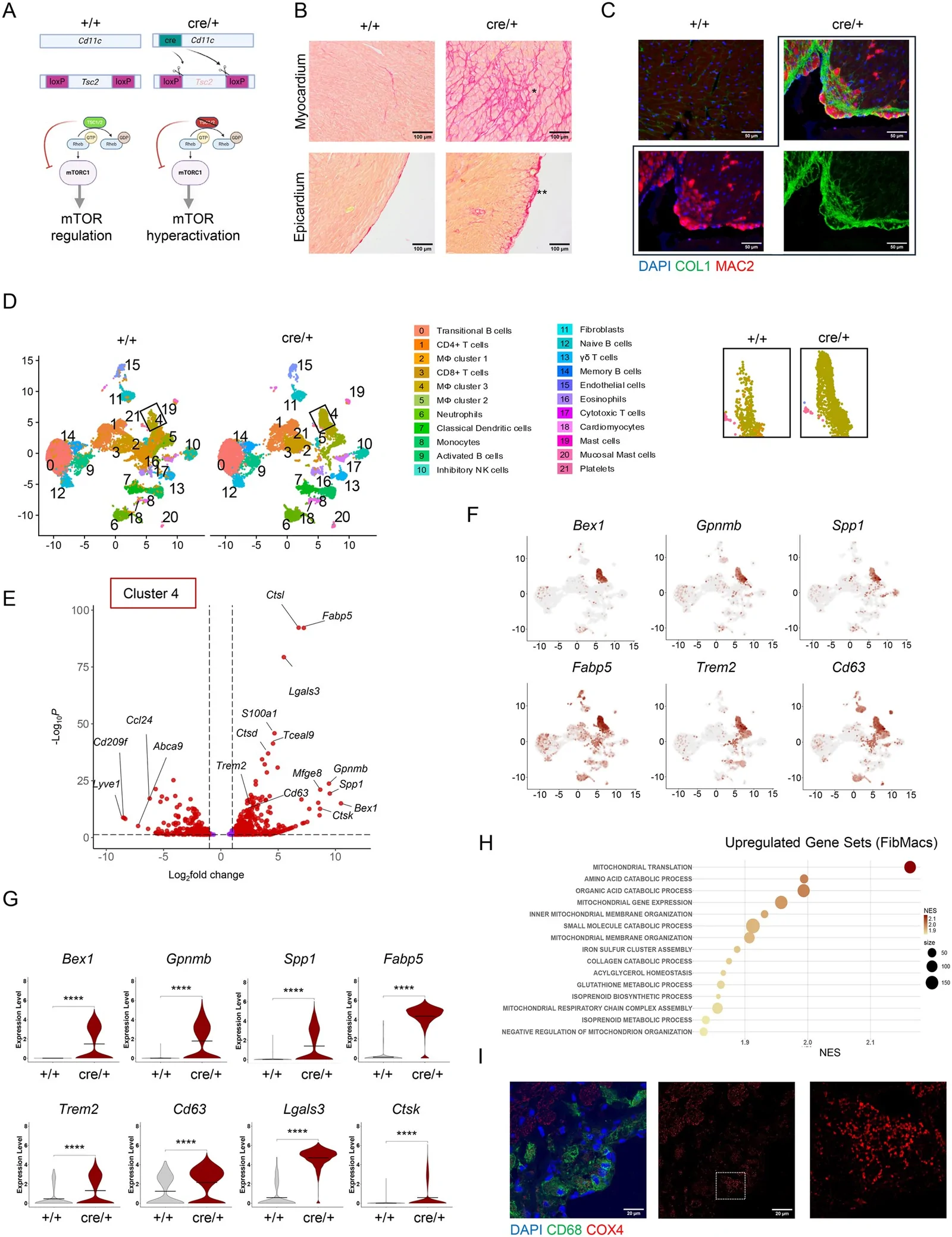
scRNA-seq reveals major changes in gene expression in macrophages. (A) Mechanism of TSC2 knockout model on mTORC1 signaling. The deletion affects only myeloid cells that express CD11c. (B) Picrosirius red staining of myocardium and epicardium showing extensive fibrosis between muscle fibers (one star) and epicardium (2 stars). (C) Immunofluorescence staining of a fibrotic scar forming around a Mac2-positive granuloma. Activated myofibroblasts produce and deposit large unorganized collagen fibers. (D) scRNA-seq of +/+ mice (*n* = 3, mean age 41.6 weeks) and cre/+ mice (*n* = 3, mean age 42 weeks) reveals 22 distinct cell populations. (E) Volcano plot of top DEGs in cluster 4 (FibMacs). (F) Uniform manifold approximation and projection showing expression of fibrogenic marker genes. (G) Expression of fibrogenic genes between control (+/+) and knockout mice (cre/+). Wilcoxon rank-sum test, ****P <.0001. (H) FGSEA results of upregulated genes in FibMacs. FGSEA analysis was performed on genes ranked by log fold change derived from pseudobulk DEG analysis. Gene sets containing 15 to 500 genes were included. *P* values were adjusted using the Benjamini–Hochberg FDR method, and pathways with FDR <0.05 were considered significant. The top 15 pathways ranked by NES are shown. (I) Immunofluorescence staining of CD68 and COX4, highlighting networks of mitochondria within granulomas (magnification 600×). Abbreviations: DEG, differently expressed gene; FDR, false discovery rate; FGSEA, functional gene set enrichment analysis; FibMacs, fibrogenic macrophages; NES, normalized enrichment score; NK, natural killer; scRNA-seq, single-cell RNA sequencing.

To investigate mechanisms linking mTORC1 hyperactivation to ECM remodeling and the resulting mechanical abnormalities, we performed scRNA-seq on whole hearts enriched for CD45^+^ cells, from cre/+ mice and control mice. Clustering of the data set revealed 22 immune cell population clusters (Figure 1D) containing three different macrophage populations (clusters 2, 4, and 5) and one monocyte population (cluster 8) (Figure S2A). Interestingly, cluster 4 had a distinct subpopulation only appearing in cre/+ mice (Figure S2B). In differentially expressed gene (DEG) analysis of the macrophage cluster 4, we identified a previously described fibrogenic gene set as the most prominent transcriptional feature of this cluster (Figure 1E). Top expressed genes were *Gpnmb*, *Spp1*, *Fabp5*, and *Bex1* (Figure 1F, G, Figure S2C). Moreover, we found strong upregulation of other fibrosis-associated genes, such as cathepsins, *Mfge8*, *Trem2*, and *Cd63* (Figure 1E). Therefore, we adopted the previously coined term “fibrogenic macrophages” (FibMacs) for this subpopulation^11^ found in CS.

Functional gene set enrichment analysis (FGSEA) of the FibMac cluster revealed strong enrichment of mitochondrial respiration, amino acid and small-molecule catabolism, aerobic respiration, and glutathione metabolism in the cre/+ mice (Figure 1H, Figure S2D), consistent with an anti-inflammatory metabolic program.^17^ Accordingly, FibMacs expressed CD206,^18^ CD163,^19^ and mitochondrial COX4 (Figure 1I, Figure S2E) and anti-inflammatory genes including *Arg1*, *Pparg*, and *Tgfb1*, whereas proinflammatory genes (*Tnf*, *Ifng*, *Il6*, *Ccl24*, *Cxcl16*) were unchanged or downregulated (Figure S2F). In addition, FibMacs exhibited a noncanonical program marked by lipid metabolism genes (*Plin2*, *Trem2*), fibrosis-associated markers (*Spp1*, *Cd63*), tissue-remodeling enzymes (*Timp2*, *Cela1*), and enrichment of collagen catabolic pathways, including multiple matrix metalloproteinases^20^^,^^21^ and cathepsins. Together, these features define FibMacs as anti-inflammatory macrophages endowed with specialized profibrotic and matrix-remodeling functions.

### FibMac emergence vastly changes the myeloid immune landscape of the heart

To better analyze subpopulations of macrophages, we isolated myeloid clusters 2, 4, 5, and 8 from the dataset (Figure S3A). We scaled the data and performed principal component analysis and dimensionality reduction, which yielded six distinct cell populations (Figure 2A, B). Now, FibMacs (cluster 0) clearly appeared as a separate cell population exclusively in cre/+ mice (Figure 2A, Figure S3B). In cre/+ mice, more than 40% of all cells were FibMacs, with monocyte numbers significantly increased as well to around 25% (Figure 2A). FibMacs highly expressed *Spp1*, *Fabp5*, *Lgals3* (MAC2), *Atp6v0d2*, *Slc6a8*, *Ctsk*, and *Gpnmb* (Figure 2C, Figure S3C). The top 30 marker genes in FibMacs (cluster 0) reflect active processes of proteostasis, lysosomal activity, lipid handling, ECM homeostasis, and immune and complement signaling (Figure 2D). Immunofluorescence staining confirmed the transcriptional findings by demonstrating strong expression of GPNMB and SPP1 in MAC2-positive macrophages (Figure 2E, F). Additionally, we found that SPP1^+^ macrophages co-localize with αSMA^+^Col1^+^ fibroblasts (Figure 2G).

**Figure 2:**
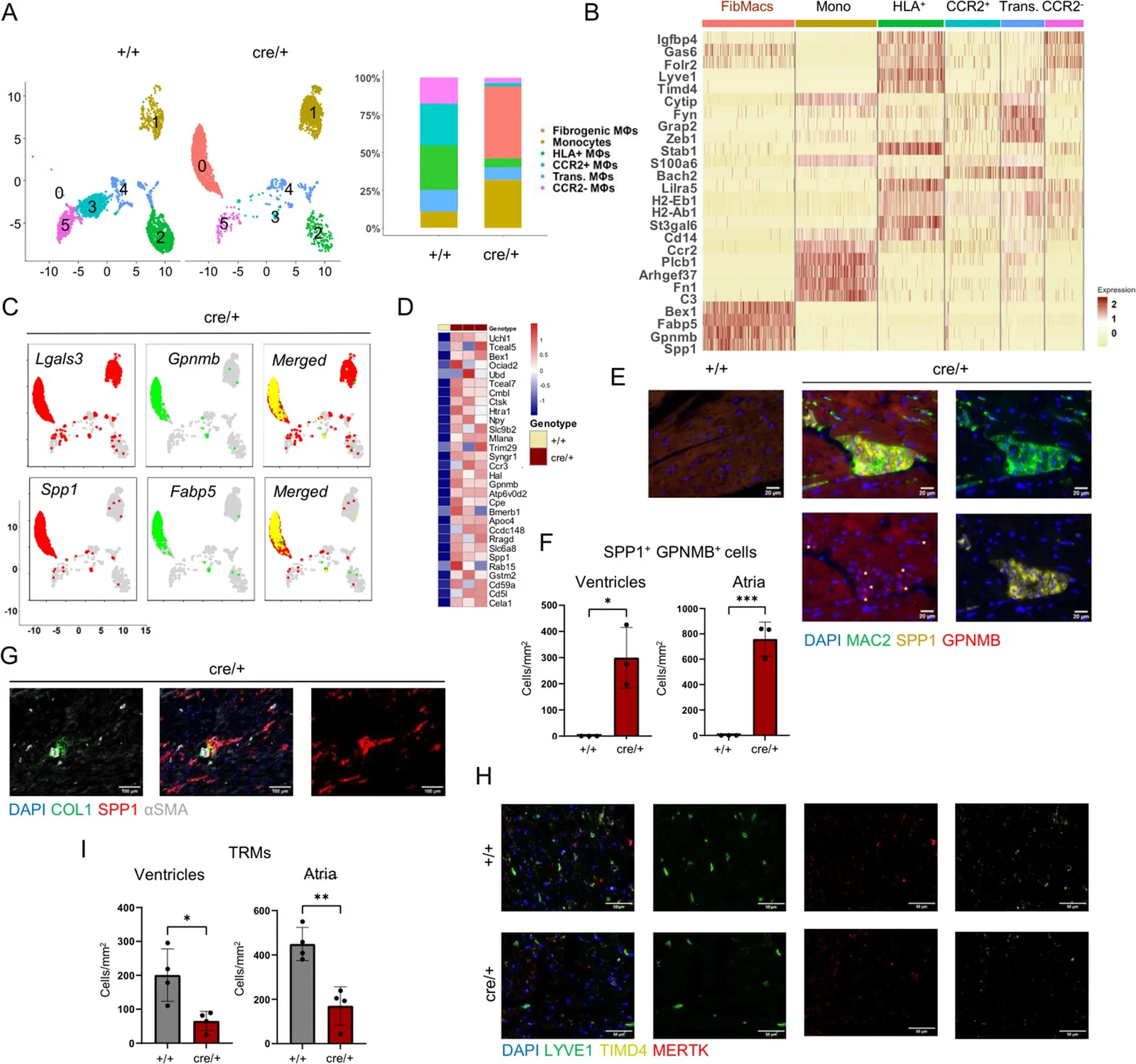
FibMac emergence vastly changes the myeloid immune landscape of the heart. (A, B) Isolating myeloid clusters yielded 6 distinct macrophage subpopulations. The composition of macrophage subpopulations is greatly changed between groups. (C) Co-expression of key FibMac genes, confirming cluster 0 as the FibMac cluster. (D) Heatmap of top 30 marker genes for cluster 0 (FibMacs); only one control sample contained cells from cluster 0 (2 cells). (E) Immunofluorescence staining of GPNMB, SPP1, and MAC2 (400× magnification). Stars indicate GPNMB^high^-expressing cells. (F) Quantification of SPP1^+ ^cells in the myocardium and atria (+/+ *n* = 3 mice; cre/+ *n* = 4 mice; mean age 38 weeks). t-test, *P <.05, ***P <.001. (G) Immunofluorescence staining of SPP1, αSMA, and Collagen 1. (H, I) Quantification of TRMs in +/+ mice (*n* = 3, mean age 46 weeks) and cre/+ mice (*n* = 3) based on co-expression of TIMD4, LYVE1, and MERTK. t-test, *P <.05, **P <.01. Abbreviations: FibMacs, fibrogenic macrophages; Mono, monocytes; TRMs, tissue resident macrophages.

We identified 3 resident macrophage populations: fetal-derived CCR2^−^ macrophages (cluster 5), which express *Lyve1*, *Folr2*, *Gas6*, and *Timd4*; HLA^+^CCR2^int^ macrophages (cluster 2) expressing various MHC-II genes; and CCR2^+^ fetal monocyte–derived macrophages (cluster 3) (Figure 2B). Interestingly, all resident populations were strongly diminished in the hearts of cre/+ mice (Figure 2A). Indeed, we verified a remarkable lack of native resident populations specifically around FibMacs and granulomas, and a generally lower density of LYVE1^+^, MERTK^+^, and TIMD4^+^ resident macrophages (Figure 2H, I).

### FibMacs originate from monocytes via TGF-β–driven differentiation

Because monocytes were markedly increased in cre/+ hearts (Figure 2A), we examined their spatial and transcriptional relationship to FibMacs. CCR2^+^ monocytes were enriched in cre/+ mice and localized at the periphery of granulomas in close contact with macrophages (Figure 3A-C). Transcriptomic analysis of monocytes (cluster 1) revealed upregulation of genes associated with migration, adhesion, and tissue retention, including *Lcn2*,^22^  *Gpnmb*,^23^ and *Itgad*^24^ (Figure 3D, E), as well as increased *Ccr2* expression (Figure S3D). Ligand–target analysis of monocyte (sender) to FibMac (target) communication identified monocyte-derived TGF-β as the ligand with the highest predicted regulatory influence on FibMac gene expression, including the target genes *Tgfbi*, *Tgfbr1*, *Csf1r*, *Hmox1*, *Alox5ap*, and *Spp1* (Figure S3E). Consistently, SPP1^+^ macrophages exhibited strong nuclear pSMAD2/3 staining, indicating active TGF-β signaling (Figure 3F). Both CCR2^+^ monocytes and SPP1^+^ macrophages expressed TGF-β (Figure 3G, H), suggesting sustained pathway activation during differentiation.

**Figure 3:**
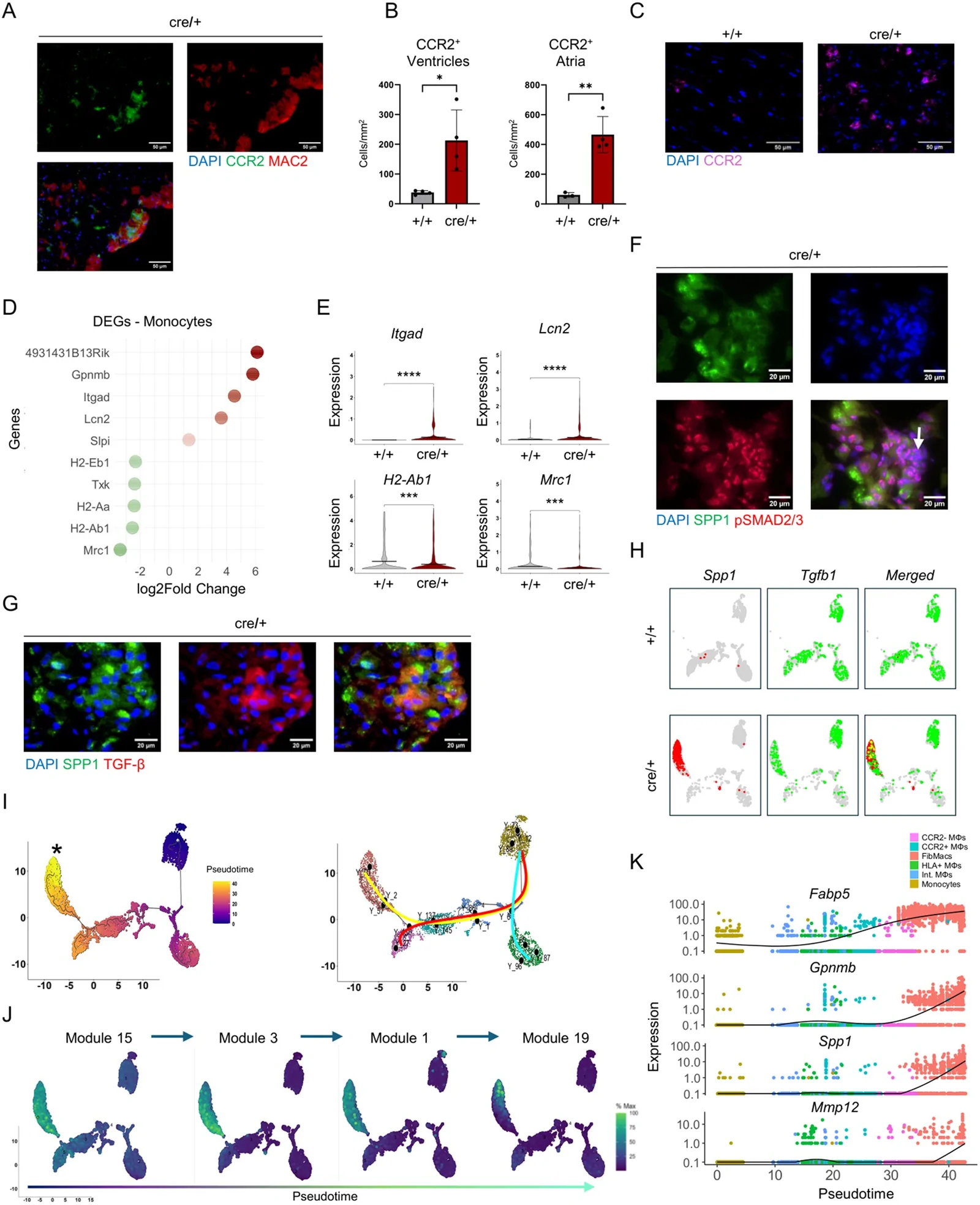
Monocytes are primed for infiltration and regulate fibrogenic macrophages (FibMacs) via TGF-β. (A) Immunofluorescence staining of CCR2 and MAC2 highlighting infiltration of monocytes around a granuloma. (B, C) Quantification and immunofluorescence staining of CCR2 between +/+ (*n* = 4, mean age 36 weeks) and cre/+ mice (*n* = 4, mean age 38.5 weeks) (200× magnification). t-test, *P <.05, **P <.01. (D) Differentially expressed genes (DEGs) in the monocyte cluster. (E) Quantification of expression levels of top DEGs. Wilcoxon rank-sum test, ***P <.001, ****P <.0001. (F) Immunofluorescence staining of SPP1 and p-SMAD2/3; arrow indicates nuclear localization of pSMAD2/3. (G) Immunofluorescence staining of SPP1 and TGF-β. FibMacs are another major source of TGF-β. (H) Uniform manifold approximation and projection (UMAP) of co-expression of *Tgfb1* and *Spp1*. (I) UMAP of cells ordered by pseudotime. Left panel shows all cells ordered by pseudotime, with the terminally differentiated state (highest pseudotime values) marked with a star. Right panel shows the three identified lineages. Of note is that the FibMac cluster is part of lineage 1 going from monocytes to the tip of the cluster (yellow line). (J) Visualization of expression patterns of the 4 fibrogenic modules. (K) Pseudotemporal gene expression of key fibrogenic genes with each dot representing a cell. The graph shows how expression changes with time, with the fibrogenic markers being expressed at later stages along lineage 1.

Trajectory inference and pseudotime analysis revealed three monocyte-derived lineages, two of which lead to resident macrophage populations (CCR2^−^ and HLA^+^CCR2^+^) and a third, unique to cre/+ mice, terminating in the FibMac cluster (Figure 3I). Using gene expression module (GEM) analysis (Figure 4A), we found monocyte differentiation regulators (*Cebpb*, *Zeb2*, and *Nfil3*) to be pro­gressively downregulated along FibMac differentiation (Figure S4B). Among all GEMs, FibMacs displayed the four most strongly correlated modules (modules 15, 3, 1, and 19), which were successively activated along pseudotime (Figure 3J). The analysis revealed sequential activation of early cytokinesis and protein assembly programs (Figure S5A, E) as well as metabolic and mitochondrial programs (Figure S5B, F), followed by an mTORC1-associated module (Figure S5C, G), culminating in a late fibrogenic module enriched for *Spp1*, *Gpnmb*, *Mmp12*, *Cela1*, *Ctsk*, and collagen catabolic enzymes (Figure 4C, Figure S5D, H). Together, these data define a TGF-β– and mTORC1–driven monocyte-to-FibMac differentiation trajectory, culminating in acquisition of a fibrogenic effector program.

**Figure 4:**
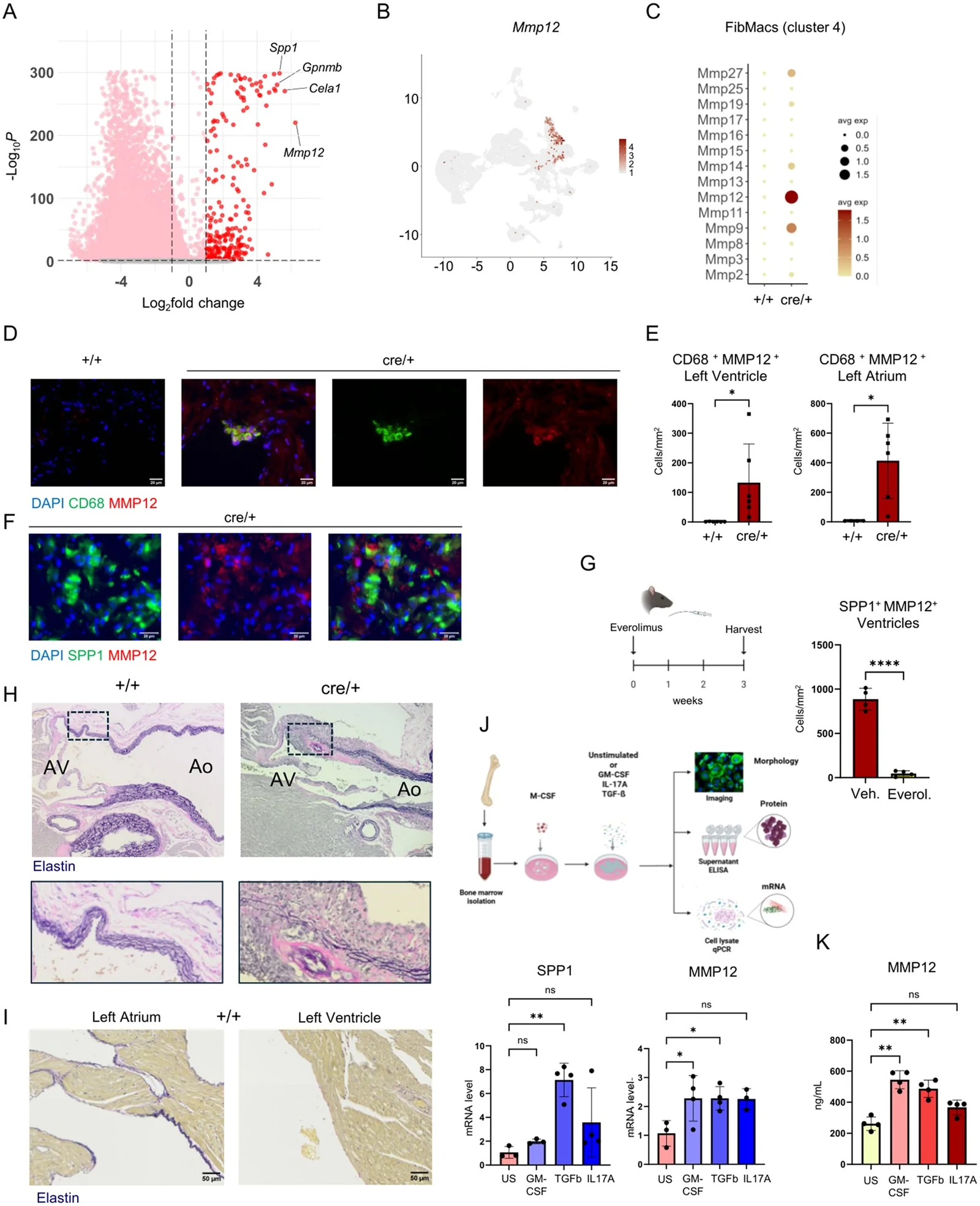
MMP12 is a key component of FibMacs. (A) Volcano plot of conserved marker genes, ie, genes expressed specifically in cluster 4 (FibMacs). MMP12 is the top conserved marker for cluster 4 (FibMacs). (B) Uniform manifold approximation and projection of *Mmp12* expression. (C) Dotplot expression profiles of all detected MMPs. Cluster 4 is the only cellular source of MMP12 in the hearts of cre/+ mice. (D) Immunofluorescence staining of MMP12 and CD68 (400× magnification). (E) Quantification of MMP12-expressing macrophage (CD68^+^) cell density in +/+ (*n* = 6, mean age 46 weeks) and cre/+ mice (*n* = 6, mean age 46 weeks). t-test, *P <.05. (F) Immunofluorescence staining of SPP1 and MMP12 (400× magnification). (G) Schematics of everolimus treatment, and quantification of SPP1 and MMP12 double-positive cells in the whole heart. Atria and ventricles analyzed together (Veh. *n* = 4; everolimus *n* = 4; mean age 41.3 weeks). MMP12-positive FibMacs were completely depleted. t-test, ****P <.0001. (H) VVG staining of elastin fragmentation in cre/+ mice. (I) VVG staining of left atrium and left ventricle. The inner chamber of the atria is lined with elastin, which is not present on the endocardium of the ventricles. (J) Quantification of qPCR results from BMDMs (+/+ *n* = 4, mean age 14.4 weeks; cre/+ *n* = 4, mean age 13.3 weeks). One-way ANOVA with Dunnett multiple comparison correction, *P <.05, **P <.01; ns, not significant. (K) Quantification of ELISA results performed on supernatants of stimulated BMDMs (+/+ *n* = 4, mean age 14.4 weeks; cre/+ *n* = 4, mean age 13.3 weeks). One-way ANOVA with Dunnett multiple comparison correction, **P <.01. Abbreviations: Ao, Aorta; Av, Aortic valve; BMDMs, bone marrow–derived macrophages; Everol., everolimus; FibMacs, fibrogenic macrophages; MMP12, matrix metalloprotease 12; US, unstimulated; Veh., vehicle; VVG, Verhoeff–Van Gieson.

### Expression of MMP12 is specifically upregulated in the FibMac population

To elucidate novel key genes that are linked to the fibrogenic gene profile in macrophages, we analyzed conserved cluster marker genes unique to the FibMac population. *Cela1*, *Spp1*, and *Gpnmb* had high increases in expression and were mostly restricted to FibMacs (Figure 4A, Tables S1 and S2). Strikingly, the top gene with the highest log_2_ fold change was *Mmp12* (Figure 4A). Expression of *Mmp12* was largely restricted to FibMacs in cre/+ mice (Figure 4B) and represented the most highly expressed metalloproteinase within this population (Figure 4C). Immunoflu­orescence confirmed MMP12 expression in CD68^+^ cells (Figure 4D). In fact, CD68^+^MMP12^+^ cells represented most of the macrophages found in the heart in the cre/+ mice (Figure 4E, Figure S6A). In addition, we linked MMP12 expression with the SPP1^+^ FibMac population (Figure 4F). Inhibition of mTORC1 signaling *in vivo* by everolimus in cre/+ mice eliminated all SPP1^+^MMP12^+^ macrophages, demonstrating an mTORC1-dependent MMP12 expression (Figure 4G, Table S3). To test for MMP12 elastolysis activity, we visualized elastic fibers with Verhoeff–Van Gieson staining and found major elastin remodeling and degradation in and around granulomas (Figure 4H, Figure S6B). In addition to myocardium, we found the largest granulomatous lesions in the atria and around the great blood vessels, where elastin is abundant (Figure 4I). Degradation of elastin by proteases creates peptide fragments termed elastin-derived peptides (EDPs).^25^ Galectin-3 (*Lgals3* or MAC2) and the elastin receptor complex (ERC)^26^ are receptors for EDPs. MAC2, as well as the 3 ERC subunits, neuraminidase 1 (*Neu1*), cathepsin A (*Ctsa*), and galactosidase 1 (*Glb1*),^27^ were expressed in FibMacs (Figure S6C), suggesting that elastin fragmentation and subsequent signaling initiated by MMP12 is involved in the fibrotic phenotype.

To evaluate whether GM-CSF, IL-17, and TGF-β, all of which have been shown to induce a fibrogenic gene programs, are also able to induce MMP12, we stimulated wild-type bone marrow–derived macrophages (BMDMs) with the cytokines (Figure 4J). qPCR analysis revealed that all 3 cytokines induced the expression of *Mmp12* but also of *Spp1 in vitro* (Figure 4J). Interestingly, TGF-β had the strongest effect on *Spp1* expression as predicted by the ligand-target gene analysis. ELISA confirmed increased macrophage secretion of MMP12 following cytokine stimulation, most prominently in response to GM-CSF and TGF-β (Figure 4K). Finally, we investigated whether we could observe the same signature in publicly available scRNA-seq datasets from murine liver fibrosis models.^11^ We found co-expression of *Mmp12* together with *Gpnmb* and *Spp1*, as well as *Tgfbr1* (Figure S6D, E in the FibMac cluster [cluster 3]). Taken together, our data strongly implicate MMP12 expression with the general fibrotic process.

### MMP12 inhibition led to decreased fibrotic lesions in the mouse heart

Our data suggested that MMP12 may play a pivotal role in driving fibrosis, prompting us to evaluate the effects of pharmacologically inhibiting MMP12 activity in our CS mouse model. We employed 2 potent and highly specific MMP12 inhibitors, linvemastat and aderamastat,^28^ to assess their therapeutic potential in mitigating fibrosis. Linvemastat (also known as FP-020) was administered orally via gavage for 3 weeks (Table S4), while aderamastat was administered twice daily for 5 weeks (Figure S7A, Table S5). Both inhibitors markedly reduced interstitial, perivascular, and epicardial fibrosis (Figure 5A-F, Figure S7B-E). Quantitative analysis revealed a marked decrease of collagen deposits in the myocardium and around blood vessels. Accordingly, the numbers of αSMA^+^ and FAPα^+^ myofibroblasts were reduced in all cardiac regions (Figure 5G-J, Figure S7F, G). The presence of granulomas and fulminant fibrosis could produce delays and abnormalities in ventricular impulse conduction. To determine whether structural improvements translated into functional recovery, we performed surface ECG analysis in mice treated with vehicle or aderamastat (Table S6). Remarkably, we found a substantial decrease in QRS duration in treated animals, compared to vehicle controls, approaching ventricular conduction time values like wild-type mice (Figure 5K). Additionally, two mice exhibited markedly shortened PQ intervals with P waves merging into the QRS complex (Figure 5L), consistent with abnormal atrioventricular conduction and an arrhythmogenic substrate in cre/+ mice.

**Figure 5:**
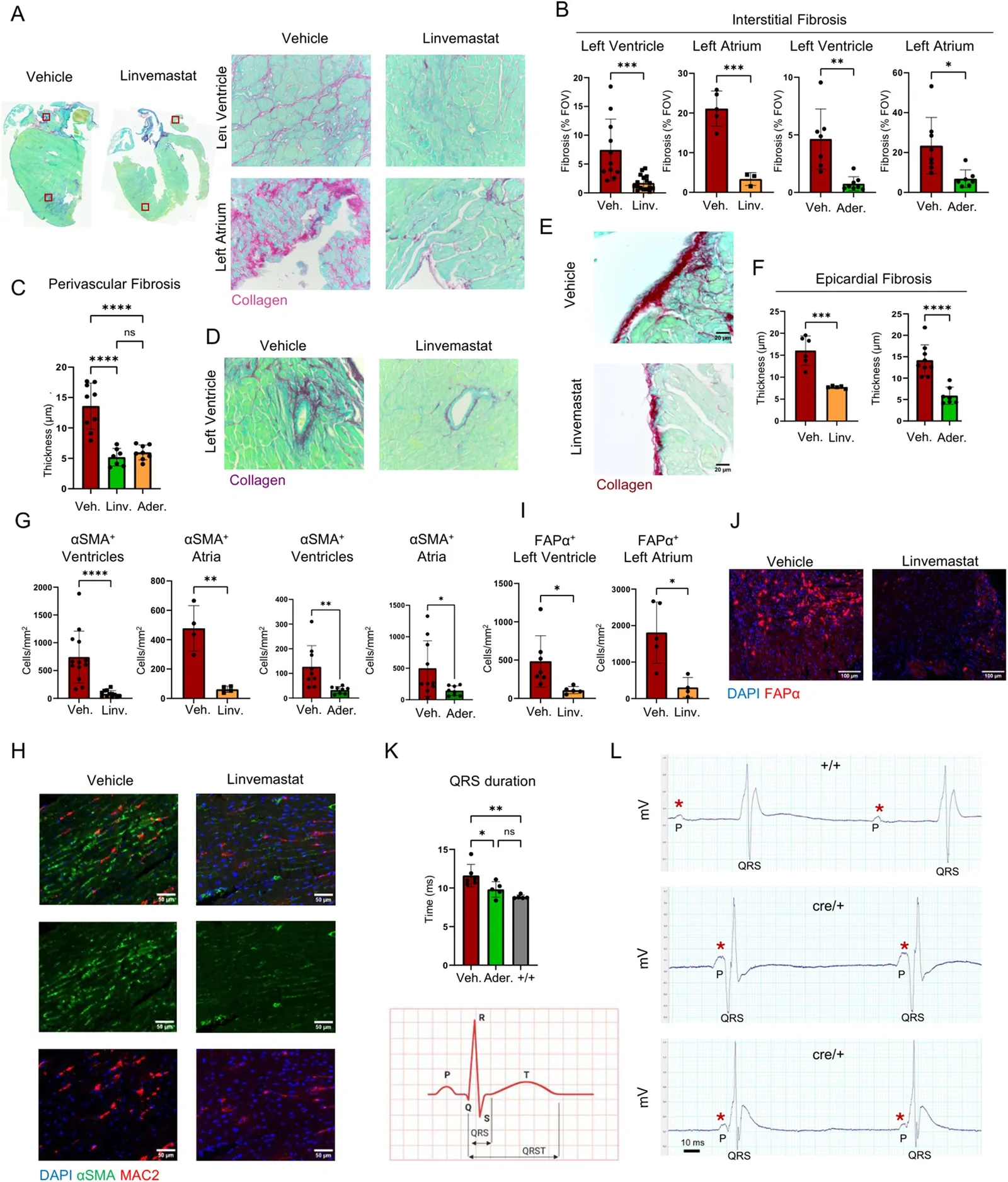
MMP12 inhibition ameliorates fibrotic burden in the heart. (A-F) Sirius red—Fast green staining and quantification of fibrotic burden (200× magnification). t-test, *P <.05, **P <.01, ***P <.001, ****P <.0001; one-way ANOVA with Tukey multiple comparison test (C). (G, H) Immunofluorescence staining and quantification of myofibroblasts after linvemastat or aderamastat treatment in the ventricles and atria (400× magnification). t-test, *P <.05, **P <.01, ****P <.0001. (I, J) Quantification and immunofluorescence staining of fibroblast activation protein (FAPα) and quantification. t-test, *P <.05. (K) Quantification of ECG after 200 mg/kg aderamastat treatment (*n* = 5, mean age 38.4 weeks) compared to vehicle-treated (*n* = 6, mean age 41.3 weeks) and +/+ mice (*n* = 5, mean age 42.2 weeks). Each data point represents the mean QRS durations from all leads of an individual mouse. One-way ANOVA with Tukey multiple comparison test, *P <.05, **P <.01; ns, not significant. (L) ECG traces (lead V1) of a control mouse (top panel; age 43 weeks) and two untreated cre/+ mice (middle and bottom panels; ages 40 and 42 weeks) showing PQ intervals that are substantially shortened such that the P-waves (stars) merge with the start of the QRS complex, leading to abnormal excitation patterns. Abbreviations: Ader., aderamastat; ECG, ­electrocardiography; FOV, field of view; Linv., linvemastat; MMP12, matrix metalloprotease 12; Veh., vehicle.

Together, these results establish MMP12 as a key effector of macrophage-driven fibrosis and demonstrate that selective MMP12 inhibition decreases myocardial fibrosis and improves cardiac electrical conduction.

### MMP12 induces and sustains FibMac differentiation and granuloma clustering

Next, we assessed whether MMP12 inhibition might directly influence granuloma architecture. Indeed, linvemastat or aderamastat treatment significantly reduced MAC2^+^ and CD68^+^MMP12^+^ macrophages across all cardiac regions (Figure 6A-C). Morphometric quantification revealed a pronounced declustering effect, particularly in the myocardium where the number of macrophage clusters (aggregates of >5 cells) and the average cluster size (number of cells in each cluster) were significantly decreased following MMP12 inhibition (Figure 6D).

**Figure 6:**
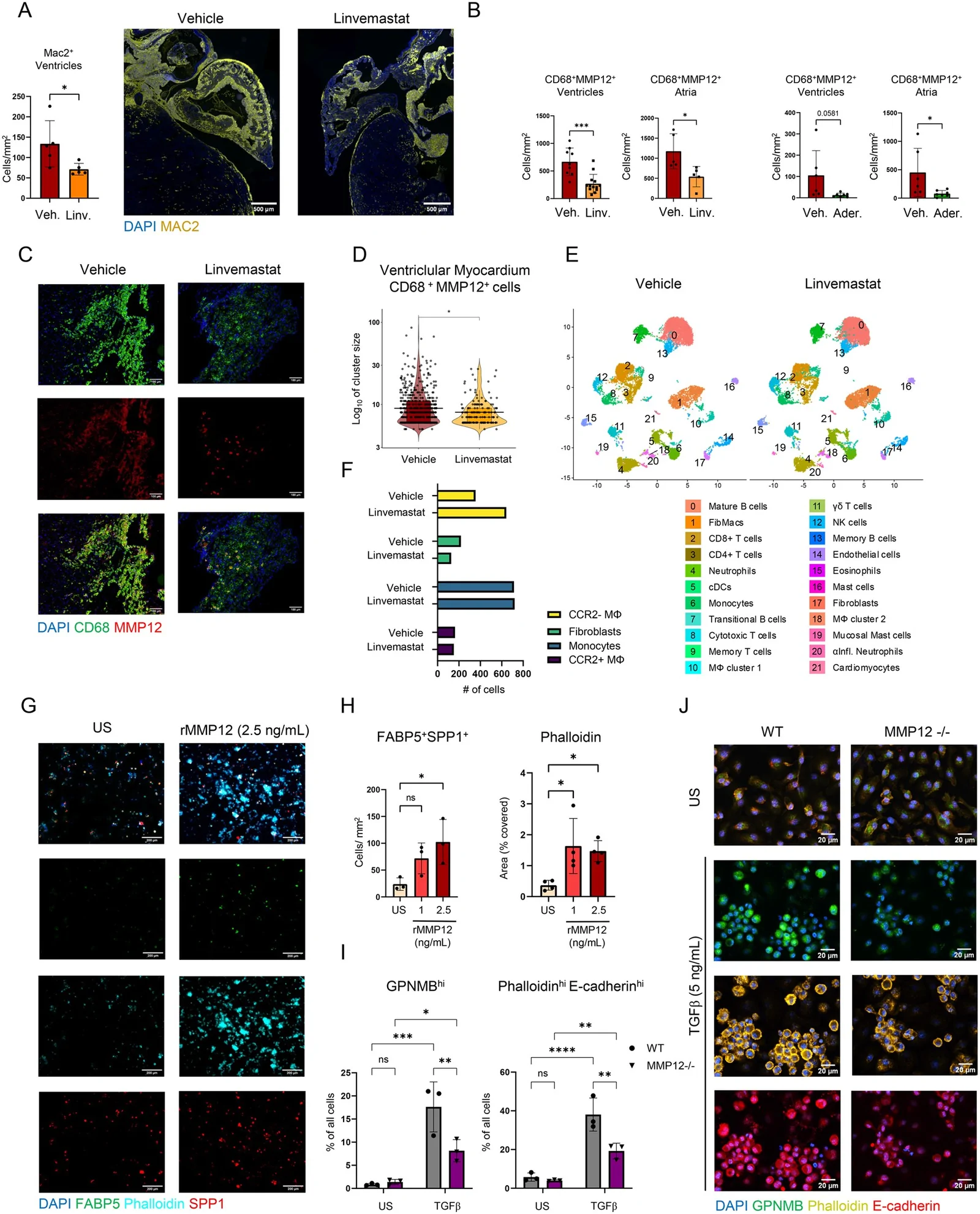
MMP12 inhibition leads to spatial and transcriptional changes in macrophages in the heart. (A) Quantification and immunofluorescence staining of MAC2^+^ macrophages after linvemastat treatment. t-test, *P <.05. (B, C) Immunofluorescence staining of CD68 and MMP12 in the left ventricle and atrium, and quantification. t-test, *P <.05, **P <.01, ***P <.001. (D) Violin plot showing cluster size (number of CD68^+^MMP12^+^ cells in cluster) in the vehicle and linvemastat treatment groups, with data being displayed on a log_10_ scale. Wilcoxon rank-sum test was used to compare the cluster size distributions between groups, with significance indicated by numerical value (significance cutoff: *P* <.05). (E) Uniform manifold approximation and projection of scRNA-seq results from vehicle- vs linvemastat-treated mice. (F) Average of cell numbers found in each sample, showing changes in subpopulations. Fibroblast numbers were halved, CCR2^−^ macrophage numbers doubled, and monocytes and monocyte-derived macrophage numbers were unchanged. (G, H) Quantification and representative images of *in vitro* stimulated WT BMDMs (*n* = 4, mean age 10.5 weeks) with two concentrations (1 ng/mL and 2.5 ng/mL) of mouse rMMP12 protein. One-way ANOVA with Šidak multiple comparison test, * *P* < .05. (I, J) Quantification and representative images of *in vitro* TGF-β–stimulated BMDMs from WT and MMP12 full-body knockout (MMP12^−/−^) mice (WT *n* = 3, mean age 19.3 weeks; MMP12^−/−^  *n* = 3, mean age 18 weeks). Two-way ANOVA, Fisher least significant different test, *P <.05, **P <.01, ***P <.001, ****P <.00001; ns, not significant. Abbreviations: BMDMs, bone marrow–derived macrophages; cDCs, classical dendritic cells; FibMacs, fibrogenic macrophages; Linv., linvemastat; MMP12, matrix metalloprotease 12; NK, natural killer; rMMP12, recombinant matrix metalloprotease 12; scRNA-seq, single-cell RNA sequencing; US, unstimulated; Veh., vehicle; WT, wild-type.

To investigate transcriptional changes in FibMacs after MMP12 inhibition, we performed scRNA-seq of whole hearts enriched for CD45^+^ cells. Bioinformatics analysis of the data set revealed 22 clusters of cells (Figure 6E). Most notably, tissue-resident CCR2^−^ macrophages were increased and fibroblasts numbers decreased, whereas monocyte and CCR2^+^ numbers were unchanged (Figure 6F). Next, we performed FGSEA analysis on the set of DEGs of the FibMac cluster 1. Here we found upregulation of microtubule cytoskeleton organization and processes related to cell cycle progression (Figure S7H), indicating a transcriptional reprogramming of FibMacs upon MMP12 inhibition.

These observations suggested that MMP12 may directly regulate FibMac differentiation. To test this, we stimulated wild-type BMDMs *in vitro* with recombinant MMP12. MMP12 induced epithelioid features, as evidenced by increased F-actin, γ-catenin, and E-cadherin expression (Figure 6G, H, Figure S7I, J), and resulted in a dose-dependent induction of the fibrogenic markers SPP1 and FABP5 (Figure 6G, H). To assess the role of endogenous macrophage-derived MMP12, we generated BMDMs from *Mmp12*^−/−^ and control mice and stimulated them with TGF-β. Genetic loss of *Mmp12* attenuated the fibrogenic response, as reflected by reduced induction of GPNMB-positive cells (Figure 6I, J). In parallel, *Mmp12* deficiency impaired the formation of F-actin–rich, E-cadherin–expressing macrophage clusters (Figure 6I, J). Consistently, pharmacologic inhibition of MMP12 in *Tsc2*-deficient BMDMs reduced phalloidin-positive F-actin structures and γ-catenin expression, indicating loss of clustering-competent, epithelioid features (Figure S7K, L). Together, these data suggest that MMP12 is sufficient to induce and is required to sustain a fibrogenic and granuloma-forming macrophage phenotype that promotes clustering, fibrotic remodeling, and granuloma expansion in this sarcoidosis mouse model.

### MMP12 drives FibMac differentiation and clustering in human CS

To assess translational relevance, we analyzed human CS samples for the presence and spatial organization of FibMac-associated proteins (Table S7). Immunofluorescence staining of Collagen 1 showed major ECM remodeling in the diseased cardiac tissue (Figure 7A). Additionally, granulomas in the septum of 6 patients with CS showed strong co-expression of MMP12 and CD68 compared to healthy samples, which had no detectable MMP12 (Figure 7B, C). We identified strong expression of SPP1 near αSMA^+^ myofibroblasts, embedded in a disorganized collagen network (Figure 7D) closely resembling the structural abnormalities seen in our mouse model.

**Figure 7:**
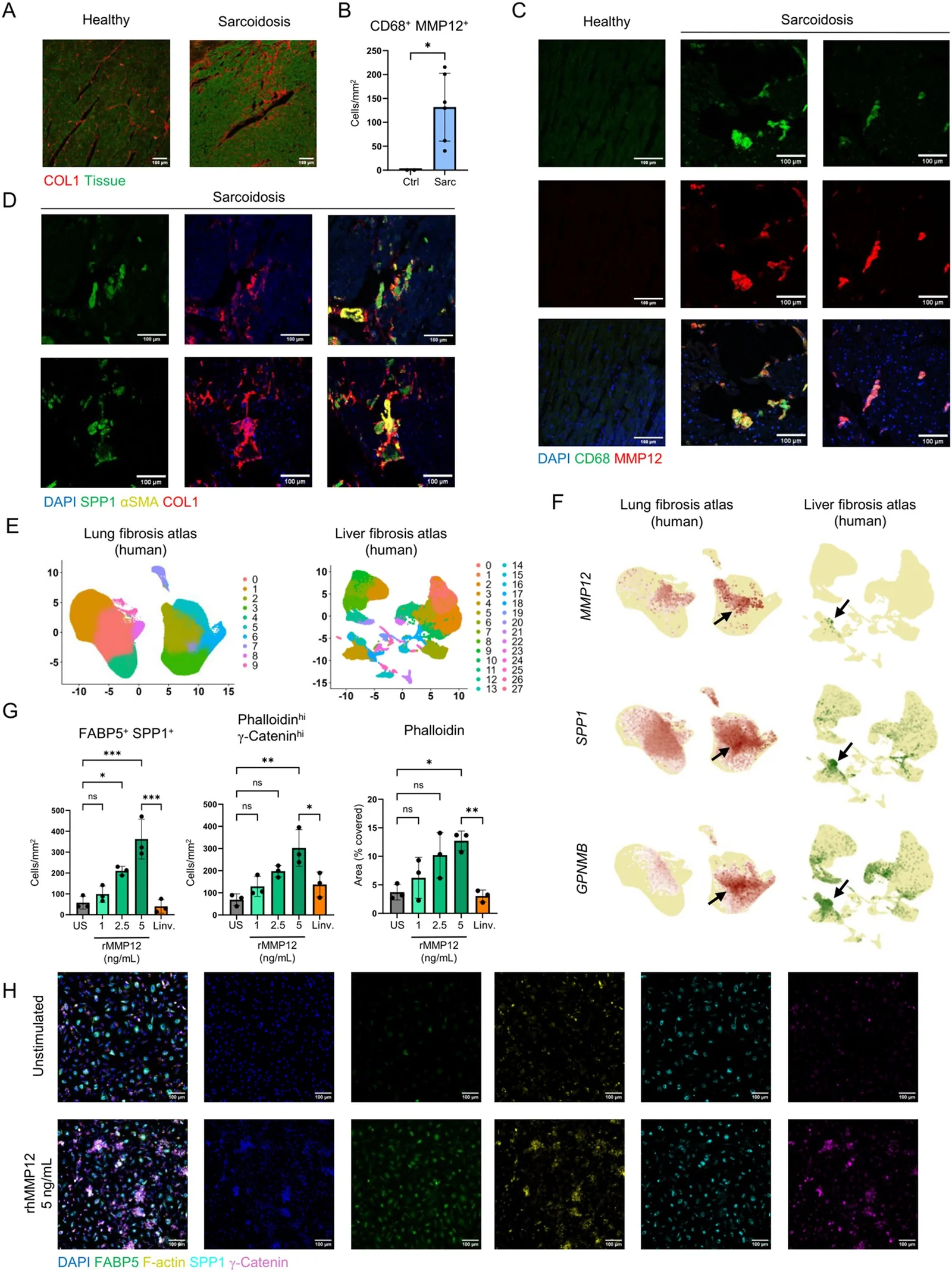
The FibMac/MMP12 signature is present in various human diseases with fibrotic characteristics. (A) Immunofluorescence staining of Collagen 1 highlighting fibrotic myocardium, septum (200× magnification). (B, C) Immunofluorescence staining and quantification of CD68 and MMP12, septum (200× magnification). t-test, *P <.05. (D) Immunofluorescence staining of SPP1, αSMA, and COL1, septum, showing fibrotic scar formation adjacent to FibMacs (200× magnification). (E, F) Uniform manifold approximation and projection of expression of key fibrogenic genes in human lung and liver fibrosis datasets. FibMac gene activity can be found in human fibrotic lung and fibrotic liver diseases. Arrows in (F) indicate FibMac clusters. (G, H) Quantification and representative images of human macrophages (*n* = 3) stimulated with rMMP12. One-way ANOVA, Tukey multiple comparison test, *P <.05, **P <.01, ***P <.001; ns, not significant. Abbreviations: FibMacs, fibrogenic macrophages; Linv., linvemastat; MMP12, matrix metalloprotease 12; rMMP12, recombinant matrix metalloprotease 12; US, unstimulated.

To examine whether the MMP12-expressing FibMacs are conserved in other fibrotic diseases, we reanalyzed previously published scRNA-seq data from patients with lung and liver fibrosis of various origins.^11^ Across datasets, we detected consistent co-expression of *MMP12*, *SPP1*, and *GPNMB* genes in FibMac clusters of individuals with disease (Figure 7E, F).

Finally, to directly test whether MMP12 induces fibrogenic reprogramming and clustering in human macrophages, we differentiated macrophages from peripheral blood mononuclear cells and stimulated them with recombinant MMP12. MMP12 stimulation robustly increased FABP5 and SPP1 protein expression and induced prominent F-actin–rich cytoskeletal structures, accompanied by increased γ-catenin^+^ and F-actin^hi^ macrophage clusters (Figure 7G, H). Preincubation with the MMP12 inhibitor linvemastat markedly attenuated both fibrogenic marker induction and clustering behavior, confirming that these effects are MMP12 dependent. The presence of MMP12^+^ FibMacs across multiple fibrotic diseases, together with their functional responsiveness to MMP12 inhibition, supports MMP12 as a macrophage-intrinsic effector and a potentially broadly applicable therapeutic target in fibrotic pathology.

## Discussion

Fibrosis arises from sustained activation of tissue repair programs in which immune and stromal cells engage in reciprocal signaling that progressively reshapes tissue architecture.^29-31^ In granulomatous diseases such as CS, fibrosis develops in close association with granulomas, yet the mechanisms that convert organized inflammatory structures into sites of irreversible scarring remain poorly defined. Our findings place fibrogenic macrophages at the center of this transition and identify MMP12 as a macrophage-intrinsic driver linking macrophage differentiation state to granuloma organization and fibrotic remodeling.

Chronic mTORC1 activation in myeloid cells establishes a permissive metabolic landscape that favors differentiation of monocytes into fibrogenic macrophages through sustained TGF-β signaling. Previous work from our group and others has demonstrated that mTORC1 signaling promotes granuloma formation and fibrosis in sarcoidosis,^16^^,^^32^^,^^33^ but how this signaling state is translated into a stable pathological cell identity remained unresolved. The present data define a differentiation trajectory in which early metabolic and mitochondrial programs precede acquisition of fibrogenic features, culminating in a late-stage program enriched for ECM remodeling enzymes and profibrotic mediators. Based on the ability of MMP12 to directly induce fibrogenic genes in macrophages, it is conceivable that sufficiently strong induction of MMP12 may partially substitute for upstream mTORC1 activity, providing an alternative route into the FibMac state under distinct inflammatory contexts.

Several genes that mark FibMacs, including SPP1 and GPNMB, have been independently associated with granulomatous inflammation and fibrosis across tissues. We now place MMP12 as a key functional node within this program that confers structural competence to FibMacs. Importantly, MMP12 is sufficient to directly induce and maintain fibrogenic gene expression, establishing it as an upstream driver of FibMac identity rather than a secondary consequence of differentiation. While FibMac differentiation is likely initiated by combined cytokine inputs such as TGF-β, GM-CSF, and IL-17A, our data indicate that MMP12 stabilizes this state, locking macrophages into a fibrogenic program. Moreover, MMP12 imposes cytoskeletal and adhesive properties characterized by F-actin remodeling and γ-catenin induction, enabling macrophages to cluster, adopt epithelioid features, and form cohesive granuloma structures. This dual function provides a mechanistic framework for how macrophages acquire a fibrogenic molecular program and, in parallel, gain the architectural capacity to organize granulomatous cell assemblies.

At the tissue level, MMP12-dependent matrix remodeling likely propagates fibrosis through multiple non–mutually exclusive mechanisms. Proteolytic processing of elastin and other matrix components generates bioactive fragments that engage pattern recognition receptors and matricellular receptors such as galectin-3 and elastokine receptor complexes, promoting leukocyte recruitment, immune activation,^34^ and fibroblast responsiveness.^35^^,^^36^ In parallel, MMP12 may shape growth factor availability and matrix stiffness, thereby reinforcing profibrotic signaling pathways. While other metalloproteinases and cathepsins are expressed within FibMacs, the dominant expression of MMP12 and the strong phenotypic impact of its selective inhibition may indicate that MMP12 occupies a rate-limiting position within this proteolytic network in chronic granulomatous fibrosis.

The chronicity of our model is an important contextual feature. Many experimental fibrosis models, including bleomycin-induced lung injury, are self-resolving and driven by acute epithelial damage,^37^ conditions under which MMP12 has been reported to be dispensable.^44^ In contrast, the *Tsc2*-deficient myeloid model develops slowly progressive, nonresolving granulomatous inflammation with accumulating fibrosis, more closely resembling CS. This chronic setting may explain why MMP12 emerges as a dominant driver, consistent with its late induction during FibMac maturation. Although previous studies found MMP12 expression in pulmonary sarcoidosis,^38^ and MMP12 has been implicated in carbon nanotube and lung fibrosis models,^39^^,^^40^ our data establish MMP12 as an important macrophage-intrinsic organizer of fibrogenic and epithelioid programs driving granuloma-associated fibrosis in sarcoidosis.

From a therapeutic perspective, current strategies for cardiac fibrosis largely target fibroblast-intrinsic pathways^41^ or broadly suppress upstream inflammatory mediators such as TGF-β, approaches that often carry substantial toxicity.^42^ The ability to attenuate granuloma integrity, reduce fibrosis, and improve cardiac electrical conduction through selective MMP12 inhibition highlights an alternative approach: targeting immune-cell effectors that organize and sustain fibrotic niches.^43^^,^^44^ MMP12 inhibition simultaneously interferes with FibMac differentiation and with their downstream matrix-remodeling effector functions. This strategy does not intervene with upstream immune activation per se but interrupts the structural and matrix-remodeling functions that lock inflammation into a fibrotic trajectory.

More broadly, our data support a framework in which FibMacs function as organizers of pathological tissue architecture, with specific proteolytic enzymes conferring architectural and fibrogenic capacity. Identifying such nodes offers a means to interrupt the progression from chronic inflammation to irreversible tissue remodeling. In cardiac sarcoidosis, MMP12 may represent such a node. The presence of related FibMac signatures across organs suggests that similar organizational principles may operate in other fibrotic diseases.

### Limitations of the study

While our data support a role for MMP12 in both granuloma organization and fibrotic remodeling, the extent to which these processes are mechanistically coupled versus representing parallel MMP12-dependent programs remains unresolved and will require future cell type–specific perturbation studies. In addition, the use of middle-aged and aged mice to model late-stage disease introduces age heterogeneity across cohorts. Although no significant differences were observed between cohorts, age-related variability in immune and fibrotic responses cannot be fully excluded. Finally, because the model is driven by chronic mTORC1 activation, the extent to which MMP12-dependent effects are contingent on sustained mTORC1 signaling remains to be fully defined.

## Supplementary Material

aamag354_Supplementary_Data

## Data Availability

This article has an online data supplement, which is accessible at the Supplements tab.
