# Supplementary material for: MMP12 induces fibrogenic macrophage differentiation to drive granuloma-associated cardiac fibrosis
Source: Am J Respir Crit Care Med. 2026 Jul 29;212(10):2409–23. doi: 10.1093/ajrccm/aamag354 (PMC13624811; doi:10.1093/ajrccm/aamag354)
Supplement: Supplementary file 1 [file aamag354_supplementary_data.zip › online data supplement.docx]

**MMP12 induces fibrogenic macrophage differentiation to drive granuloma-associated cardiac fibrosis**

ONLINE DATA SUPPLEMENT

**SUPPLEMENTAL METHODS**

Study Design

This study was designed to define macrophage-intrinsic effector mechanisms linking granulomatous inflammation to fibrotic remodeling in cardiac sarcoidosis. We employed a mouse model of cardiac sarcoidosis in which myeloid-specific deletion of Tsc2 results in chronic mTORC1 activation, spontaneous granuloma formation, and progressive cardiac fibrosis across myocardial regions. Single-cell RNA sequencing of cardiac immune cells was performed to identify fibrogenic macrophage populations and delineate mTORC1-associated differentiation trajectories. Bioinformatic findings were validated by immunofluorescence and histopathology. The contribution of macrophage-derived MMP12 to fibrotic remodeling was examined by assessing cell-type–specific MMP12 expression and elastin fragmentation *in vivo* using Verhoeff–van Gieson staining. In parallel, *in vitro* stimulation of murine bone marrow–derived macrophages from wild type, Tsc2^fl/fl^CD11c^cre/+^, and MMP12^-/-^ mice and human monocyte-derived macrophages with recombinant MMP12 was used to determine whether MMP12 directly induces fibrogenic macrophage differentiation and clustering. To determine functional relevance, two selective MMP12 inhibitors were administered to transgenic mice and age- and sex-matched controls, and effects on granuloma architecture and fibrosis were quantified. Animals were age- and sex-matched to experimental groups. Experiments were performed in batches with comparable results, and representative batch data are shown as specified in figure legends. Sample sizes were chosen according to approved animal ethics protocols. The number of animals and statistical tests are provided in the respective figures. Both male and female mice were studied with similar outcomes. Translational relevance was assessed by immunohistochemical analysis of human cardiac sarcoidosis tissue and by reanalysis of publicly available transcriptomic datasets.

Human Participants

Formalin-fixed, paraffin embedded (FFPE) human heart tissue samples were obtained postmortem from the interventricular septum through the Cardiac Risk of the Young Centre for Cardiac Pathology at St. George’s University of London. A total of eight specimens were used in the study: six from individuals with confirmed diagnosis of cardiac sarcoidosis and two from healthy controls. Cardiac sarcoidosis was determined as the cause of death based on comprehensive autopsy evaluations performed by an expert cardiac pathologist. The sarcoidosis group included four males and two females, aged 33 to 48 years (median age 38), with varying comorbidities including asthma (n=1), tuberculosis (n=1), and fibromyalgia (n=1), while three had no documented co-existing conditions. The control group consisted of a 35-year-old male who died by suicide and a 30-year-old female who died from traumatic injury; both had no known cardiac pathology. All tissues were fixed in 10% neutral buffered formalin and processed into paraffin blocks under standardized pathology protocols. Serial tissue sections from these blocks were used for immunohistochemical analyses in this study.

Mouse model

C57BL/6J mice carrying loxP-flanked (floxed) alleles of the *Tsc2* gene (Tsc2^fl/fl^) were crossed with mice expressing Cre recombinase under the control of the *Itgax* promoter (*Itgax*^cre/+^; CD11c^cre/+^) to generate CD11c^cre/+^; Tsc2^fl/fl^ mice (hereafter referred to as cre mice, denoted as cre/+) and CD11c^+/+^; Tsc2^fl/fl^ littermate controls (referred to as +/+ mice). MMP12 full-body knock-out (MMP12^-/-^) mice and control mice were purchased from The Jackson Laboratory and bred at the Medical University of Graz. All other mice were sourced from the Department of Laboratory Animal Science and Genetics, Himberg, Austria. All mice were housed in a specific pathogen-free in-house animal vivarium under controlled conditions (ambient temperature ~22°C, 12-hour light/dark cycle, standard rodent chow and water provided ad libitum). Animals of both sexes were used, and experimental groups were matched for age and sex. No substantial sex-specific differences were observed in phenotypes or treatment response during the course of experiments.

Electrocardiography (ECG)

ECG recordings were obtained from mice under isoflurane anesthesia (4 vol% for induction, 1.5 vol% for maintenance). Six needle electrodes were placed subcutaneously in standard limb lead configuration and precordial lead positions V1, V6, and V6R. Signals were digitally acquired using the ML183 Octal Bio Amp System (ADInstruments, Oxford, UK). ECG analysis was performed on averaged traces (200 beats) using LabChart Pro 8 (ADInstruments) and custom-designed analysis software. QRS duration was measured from the onset of ventricular depolarization to the latest sharp deflection. Comparisons between +/+ and cre/+ mice were performed in 11 animals (5 +/+ and 6 cre/+). ECG measurements in treated cohorts were obtained from 11 mice (6 vehicle-treated and 5 Aderamastat-treated). Mice were analyzed 24 hours after the final administration of Aderamastat (200 mg/kg) or vehicle.

Linvemastat preparation

Linvemastat was acquired from Foresee Pharmaceuticals (Taipei, Taiwan) in powder form. The drug was directly diluted in distilled water to the desired working concentration of 30 mg/kg. Distilled water has been used as a vehicle control.

Linvemastat treatment

A total of 32 *CD11c*^cre/+^, *TSC2*^fl/fl^ mice (mean age, 50.1 weeks) were randomly assigned to receive with Linvemastat (30 mg/kg) or with vehicle control via oral gavage, once daily for 21 consecutive days. Both male and female mice were included, with no observed sex-related differences in response. Following the final dose, mice were anesthetized and euthanized. Hearts were rapidly excised, washed in ice-cold 1x PBS, and fixed overnight in Histofix (Carl Roth, P087.3). Tissues were subsequently processed into paraffin-embedded blocks for downstream histological analysis

Aderamastat preparation

Aderamastat was acquired from Foresee Pharmaceuticals in powder form containing 25% of active substance. A polymeric solubilizer and matrix forming polymer – Soluplus™ (BASF Pharma, Germany) was used to increase the solubility of the drug. Powdered Soluplus™ was mixed with ddH_2_O to create a 1% solution. The resulting solvent was added in increments to the drug powder, to create an emulsion with a pestle and mortar with a stock concentration of 30 mg/mL. The stock solution was diluted with vehicle to the desired working solution of 100 mg/kg. Pure Soluplus™ solution was used as a vehicle control.

Aderamastat treatment

Twenty *CD11c*^cre/+^, *TSC2*^fl/fl^ mice (mean age, 35 weeks) were treated with Aderamastat (100 mg/kg) or vehicle twice daily (morning and evening) via oral gavage for 5 weeks. After the final dose, mice were anesthetized and euthanized. Hearts were isolated, rinsed in ice-cold 1xPBS, and embedded in OCT compound using cryomolds placed on dry ice. Frozen heart samples were stored at -80°C until further processing for cryosectioning.

Everolimus preparation and treatment

Everolimus powder (Seleckchem, RAD001) was formulated in a vehicle solution composed of 1x PBS with 0.05% DMSO (D2438, Sigma Aldrich), 30% polyethylene glycol or PEG (Sigma Aldrich, 202 371), and 5% Tween-80 (Sigma Aldrich, P1754-25mL). Male and female *CD11c*^cre/+^, *TSC2*^fl/fl^ mice (mean age 26 weeks) received daily oral gavage of everolimus (5 mg/kg) or vehicle for 3 weeks. Mice were sacrificed following the final treatment. Hearts were harvested, fixed overnight in Histofix, and processed into paraffin-embedded blocks for histological analyses.

Immunofluorescence staining

We used 5 µm thick paraffin sections cut on a microtome and dried overnight, or 5 µm thick cryosections sectioned with a cryostat, for stainings. Deparaffinization was performed in Neo-Clear™ (Merck, 1.09843) an aliphatic hydrocarbon mixture solution, and a series of ethanol washes (96% and 100%), and rehydrated in dH2O and 1x PBS. Heat-induced epitope retrieval was performed at pH 6 antigen retrieval solution (Thermo Scientific, 00-4955-58) in boiling solution for 30 minutes. Permeabilization was done in 1x PBS with 0.3% Triton X. Both paraffin and cryo sections were blocked in serum and incubated overnight with a primary antibody cocktail. The following day, sections were washed in 1x PBS containing 0.025% Triton-X (Sigma, X100) and incubated with a fluorescently labelled secondary antibody mixed with DAPI nuclear stain solution (Merck, D9542) for 1 hour at room temperature. Slides were then washed, mounted and imaged. Primary antibodies used were: rat anti-mouse CD68 (Biolegend, 137002, 1:100), rat anti-mouse MAC2 (Cedarlane, CL8942AP, 1:800), goat anti-mouse SPP1 (R&D, AF808, 1:100), rabbit anti-mouse GPNMB (abcam, ab188222, 1:100), rabbit anti-mouse/human MMP12 (Bioss, bs1854R, 1:100), rabbit anti-mouse pS6 (240/244) (CST, 5364, 1:500), rabbit anti-mouse CCR2 (abcam, ab273050, 1:200), TIMD4 (Lifespan Biosciences, LS-B1413, 1:100), rabbit anti-mouse CD163 (Bioss, bs-2527R, 1:100), goat anti-mouse MERTK (R&D, AF591, 1:200), rat anti-mouse LYVE1 (Invitrogen, 14-0443-82, 1:100), goat anti-mouse CD206 (Invitrogen, PA5-46994, 1:200), mouse anti-mouse TGFβ1 (Invitrogen, MA5-16949, 1:100), rabbit anti-mouse pSMAD2/3 (Ser465, Ser467, Ser423, Ser425) (Invitrogen, PA5-110155, 1:100), rabbit anti-mouse FAPα (abcam, ab218164), mouse anti-human/mouse αSMA (DAKO, M0851, 1:200), rabbit anti-mouse Collagen 1 (CST, 72026, 1:100), goat anti-mouse E-Cadherin (R&D, AF748, 1:200), rat anti-mouse γ-Catenin (abcam ab184919, 1:100), Phalloidin AF555 (Invitrogen, A30106, 1:800), mouse anti-mouse/human FABP5 (Biolegend, 613951). Secondary antibodies used were donkey anti-rat AF555 (abcam, ab150154, 1:500), donkey anti-rat AF488 (Invitrogen, A21208, 1:500), donkey anti-goat AF488 (Invitrogen, A32814, 1:500), donkey anti-rabbit AF647 (Invitrogen, A32795, 1:500), donkey anti-mouse AF555 (Invitrogen, A31570, 1:500). Antibodies used for staining of human tissue were rabbit anti-human Collagen1 (CST, 72026, 1:100), rat anti-human CD68 (Invitrogen, 14-0681-82, 1:200), rabbit anti-mouse/human MMP12 (Bioss, bs1854R, 1:100), goat anti-human SPP1 (AF1433), mouse anti-human/mouse αSMA (DAKO, M0851, 1:200) rabbit anti-human γ-Catenin (Biolegend, 624051, 1:100), Phalloidin AF555 (Invitrogen, A30106, 1:800), mouse anti-mouse/human FABP5 (Biolegend, 613951). Secondary antibodies used were donkey anti-rabbit AF647 (Invitrogen, A31573, 1:500), donkey anti-rat AF488 (Invitrogen, A21208, 1:500), donkey anti-goat AF488 (Invitrogen, A11055, 1:500), donkey anti-mouse AF555 (Invitrogen, A31570, 1:500), donkey anti-mouse AF488 (Invitrogen, A11029, 1:500), donkey anti-goat AF647 (Invitrogen, A32849, 1:500), goat anti-rabbit AF750 (Invitrogen, A21039, 1:500).

Hematoxylin & Eosin

7 µm thick tissue sections were deparaffinized and rehydrated as described above. After a brief rinse in distilled water, tissue sections were incubated with Hematoxylin (Sigma Aldrich, MHS32-1L) for 3 minutes. Afterwards, slides were washed in running tap water, differentiated with two dips in HCl-based differentiation solution (Sigma Aldrich, A3179), washed in tap water, and placed in Scotts Tap water substitute. Afterwards, slides were placed in Eosin + Phloxine (Sigma Aldrich, HT110332-1L) for 30 seconds. After a brief rinse in water, the slides were dehydrated in ethanol and Neo-Clear™ (Sigma Aldrich, 1098435000) and mounted in a resinous mounting medium Neo-mount™ (Sigma Aldrich, 109016).

Picrosirius Red & Sirius Red- Fast Green staining

7 µm thick tissue sections were deparaffinized and rehydrated as described above. Cryosections were cut at 10µm thickness on a cryostat. After a brief rinse in distilled water, deparaffinized tissue sections were incubated with Sirius Red – Fast green staining solution (Chondrex, 9046) or with Picrosirius Red (abcam, ab246832) for 1 hour, to stain collagen fibers red, cytoplasm and extracellular matrix green or yellow, and erythrocytes yellow. Two washes in acidified water (0.5% Acetic acid) were performed to rinse off excess staining solution. Sections were dehydrated in 96% and 100% ethanol and cleared with Neoclear™ and subsequently mounted with Neo-mount™ and dried at room temperature until imaging.

Verhoeff van Gieson elastic staining

Deparaffinization and rehydration of 5 µm thick sections were performed as described above. For elastin visualization a Verhoeff van Gieson (VVG) Elastica staining kit was used (Morphisto, 18553) as per manufacturer’s instructions. Slides were placed in Verhoeff’s solution for 15 min, after which they were manually, one by one, differentiated in Ferric chloride. After washing off the excess with ddH_2_O and rinsing in ethanol, the slides were incubated in van Gieson Picrofuchsin for 2 min. Slides were dehydrated in 96% and 100% ethanol, cleared in Neo-Clear™, and mounted with Neo-mount™.

Immunocytochemistry

Fixed cells were washed with 1X PBS and blocked and permeabilized in 1x PBST (0.3%) 2.5% donkey serum (Thermo Fisher, A44638) for 40 minutes. After 3 washing steps, 2 minutes each, Cells were incubated overnight with a primary antibody cocktail at 4°C. On the next day, cells were washed in 1X PBS, and incubated for an hour with secondary antibody solutions, containing 1X DAPI, and Phalloidin. Cells were carefully washed and stored in 1X PBS. Cells were not mounted for imaging.

Human macrophage differentiation from peripheral blood mononuclear cells (PBMCs)

Human peripheral blood mononuclear cells (PBMCs) were obtained from healthy adult volunteers according to national law and approved by the ethics committee of the Medical University of Vienna (ECS 1198/2021). Written informed consent was obtained from all donors in accordance with the Declaration of Helsinki. PBMC were isolated from fresh blood from healthy donors with informed consent (n=3, mean age = 32 y) by using density gradient centrifugation. Collected blood was mixed with an equal amount of warm RPMI-1640 media with 2 mM Glutamine (Gibco, 11875-093). 35 mL blood-media was carefully added on top of was placed over a 15 mL layer Lymphoprep density gradient medium (StemCell, 18061) and centrifuged at 8000g for 20 minutes (acceleration = max, deacceleration = 0). The resulting cloudy layer of PBMCs was gently removed from the falcon, centrifuged, and the pellet washed in 5 mL of 1X PBS 3 times. Cells were counted and diluted in RPMI-1640 media with 2 mM Glutamine (Gibco, 11875-093), containing 10% fetal bovine serum (Gibco, 10082-147), 1% Penicillin-Streptomycin (Sigma, P4333-100ML), and 20 ng/ml of M-CSF (Biolegend, 574804) to reach a concentration of 1x10^6^ cells/mL. Cells were plated on 6 cm Polystyrene dishes (Falcon, 351007), on days 3 and 5, 2mL of fresh media were added.

*In vitro* stimulation of human macrophages

Macrophages were harvested on day 6 using 5 mL of Accutase cell detachment medium (Invitrogen, 00-4555-56) with gentle scrapping. Cells were centrifuged, counted, seeded on a 96-well µ-Plate (ibidi, 89626), and either stimulated with 1, 2.5 or 5 ng/mL of recombinant human MMP12 (Biolegend, 764202), prestimulated with 50 µg/mL Linvemastat for 1 hour before addition of 5 ng/mL rMMP12, or left unstimulated. After 24 hours, the cells were washed in 1X PBS, fixed with Histofix, washed and stored at 4°C until immunocytochemistry stainings.

Bone marrow derived macrophage (BMDM) generation

Mice were euthanized and the hind legs were isolated. The muscles around the hip joint and knee joint were carefully removed and the Achilles tendon was cut to expose the bones. Then the limb was carefully pulled out of the hip joint. Isolated bones were placed in 1x cold PBS and kept at 4°C. Under a laminar flow hood, the femur and tibia were separated, and the bones were washed in 70% ethanol for 20-30s and then transferred in 1X PBS in a petri dish. The epiphysis was cut off on both ends and the bones were flushed with a 27G needle onto a 100 µm strainer placed on a 50 ml Falcon tube. The bones were flushed until they were completely white. The collected marrow was centrifuged at 500g for 5 minutes to create a pellet, and the supernatant was discarded. Afterwards, the pellet was resuspended in 1ml of 1x red blood cell lysis (RBC lysis) solution (Biolegend, 420301) and incubated for 5 minutes. To stop the reaction, 9 ml of 1x PBS were added to the cells. Cells were centrifuged at 500g for 5 minutes, and the pellet was resuspended with 1 ml of RPMI-1640 media with 2 mM Glutamine (Gibco, 11875-093), containing 10% fetal bovine serum (Gibco, 10082-147), 1% Penicillin-Streptomycin (Sigma, P4333-100ML), and 30 ng/ml of M-CSF (Biolegend, 576406). The total number of cells was determined using a Countess 3 automatic cell counting device (Thermo Fisher Scientific) using Trypan blue staining to visualize cells and evaluate viability. Next, cells were diluted with media to reach a concentration of 1x10^6^ cells/ml. Cells were carefully resuspended and seeded in 150 mm tissue culture plates. After three days, 3 ml of fresh medium supplemented with 30 ng/ml M-CSF were added until a layer of adherent and well differentiated cells was covering around 80-90% of the surface. On the fifth day the media was removed, and cells were briefly washed with 1x PBS. Enzyme free dissociation solution (Merck, S-014B) was added to the plates and incubated for 10 minutes to detach the BMDMs. Cell scrapers were used to aid in the removal of any adherent cell clusters. The collected cells were spun down at 500g for 5 minutes and resuspended in 1 ml of media and cell count was determined using Trypan blue as previously described. Next, BMDMs were seeded in a 12-well plate with each well containing 1x10^6^ cells.

*in vitro* stimulation

On the fourth day, cells were detached using an enzyme free dissociation solution (S-014-B, Merck Milipore) and counted using an automated cell counter (Countess 3, Thermo Fisher) using trypan blue to assess cell viability. Cells were seeded in 12-well or 96-well plates, with each well containing 1x10^6^ and 1.5x10^5^ cells, respectively. Cells were then left to adhere for 4 hours, after which non-adherent cells were washed off. BMDMs were stimulated with either GM-CSF (30 ng/mL; Biolegend, 315-03-100UG), TGFβ (5 ng/mL; Biolegend, 763102), or IL-17 (20 ng/mL; Peprotech, 210-17-5UG), or MMP12 (1-5 ng/mL, abcam, ab316590) at 37°C in a humidified 5% CO_2_ incubator. As a control condition, unstimulated cells were used on which RPMI media++ supplemented with 5% FBS and 1& Penicillin-Streptomycin was added. Experiments with Linvemastat (50 µg/mL in 1X PBS) were performed by pretreating the cells with the inhibitor prior to stimulation. After 24 hours, supernatants were collected, spun down, and stored at -80C.

RNA isolation

To extract RNA from the cells, the RNeasy Mini isolation kit (Qiagen, 74104) was used as per the manufacturer’s instructions. In short, a silica membrane-based spin column method was used. After collecting the supernatants, cells were lysed and ethanol was added, then the lysate was centrifuged. The membrane was washed with buffers and centrifuged to remove any protein and DNA residuals from membrane. Finally, the lysate was eluted into nuclease-free water and isolated RNA was stored at -80°C until further processing.

cDNA synthesis and qPCR

RNA concentration was measured photometrically using a NanoPhotometer NP80 (Implen, Germany). From each sample 100 ng of RNA were used to produce complementary DNA (cDNA) using a GoScript™ Reverse Transcriptase kit (Promega, A2791) as per manufacturer’s instructions. cDNA was diluted 1:10 in nuclease-free water and amplified using the GoTaq® qPCR Master Mix kit (A6001, Promega), using SYBR Green as a reporter, as per manufacturer’s instructions. Samples were analyzed using a StepOnePlus™ Real-Time PCR system (Thermo Fisher, 4376600). The relative gene expression of *Spp1* and *Mmp12* was normalized to β-actin expression using the ∆∆Ct method, and log2 transformed ∆∆Ct values were plotted for data visualization. The sequence of the primers used are as follows: *Spp1* forward: TCGGAGGAAACCAGCCAAGGACT, reverse: AGGCTTCTTCTCCTCTGAGCTGCCA; *Mmp12* forward: CACACTTCCCAGGAATCAAGCC, reverse: TTTGGTGACACGACGGAACAGG; *Actb* forward: CCAGCCTTCCTTCTTGGGTA, reverse: CTTTACGGATGTCAACGTCACAC.

Enzyme-linked immunosorbent assay (ELISA)

To quantify MMP12 protein levels in cell culture supernatants from stimulated and unstimulated BMDMs we used a SimpleStep ELISA® kit (abcam, ab246540) as per the manufacturer’s instructions. In short, supernatants were diluted 1:150 in provided sample buffer and added to the plate. In parallel, a serial dilution of recombinant MMP12, provided in the kit, was done to obtain standard curve values, ranging from 8000 pg/mL to 125 pg/mL. Afterwards, an antibody cocktail containing a capture and detection antibody was added to the samples and standards and incubated at room temperature for 60 minutes. After washing in provided wash buffer, wells were developed using Tetramethylbenzidine (TMB) for 8 minutes. The reaction was stopped using a provided stop buffer, and absorbance was measured at 450 nm on a Biotek Synergy HTX plate reader. Blank values were subtracted from sample and standard absorbances, and sample concentrations were determined based standard curve interpolation.

Microscopy and image analysis

All images were acquired on an Olympus microscope, model BX63 (Evident, Germany), or on an inverted light microscope APEX, model APX100 (Evident, Germany). Whole-tissue overviews were taken at 100x magnification for fluorescence images, and at 200x brightfield magnification for Sirius Red- Fast Green images. Representative images were acquired at 400x and 600x magnification. Imaging of human sarcoidosis and control samples was done on a Nikon A1R confocal microscope at 200x magnification, at the Imaging facility of the Cardiovascular & Genomics Research Institute, St George’s University of London. Image analysis was performed in QuPath v0.4.4. Analysis of Sirius Red – Fast green images were performed by selecting fields of view of equal size of fibrotic regions in vehicle treated animals and comparing them to fields of view of corresponding anatomical structures in the treated groups. Fibrotic structures with positive Sirius red staining were expressed as % of Area covered of each field of view. A medium-high threshold was used to define dense positive staining. Immunofluorescence images of tissue sections were manually segmented into anatomical regions and the tissue vs background boundaries defined by the average signal intensity of all acquired channels, resulting in precise tissue segmentation. TRM populations were evaluated based on 4-5 randomly chosen heart regions, from ventricles and atria, taken with a 400x objective. All images from *in vitro* experiments were evaluated using the same downstream analysis protocol. In short, all cells were detected using a watershed algorithm on the DAPI channel. To differentiate positive from negative cells, a Random Trees network was trained in a semi-supervised way to detect positive cells based on signal intensity of either a combination of channels, or on a singular channel alone. The algorithm was manually annotated until a sufficient degree of positive/negative classification was achieved. The trained network was saved and applied to all images from the same experiment. Cell type abundance was expressed as number of positive detections normalized to total area, or as a percentage of the total population. DBSCAN (Density-Based Spatial Clustering of Applications with Noise) was applied in QuPath v0.4.4. to identify CD68^+^ MMP12^+^ cell clusters based on centroid coordinates using a distance threshold of 5 µm and minimum of 5 cells (smaller clusters were excluded). Each cell was assigned to a cluster ID and cluster size, which were recorded as measurements.

Single cell suspension and fluorescence aided cell sorting (FACS)

Hearts were harvested and placed on ice cold PBS before further processing. The tissue was minced and digested with 450U/ml Collagenase I (Sigma, C0130), 60U/ml DNase I (Sigma, D4513), and 60U/ml Hyaluronidase (Sigma, H3506) for 45 minutes at 37°C on a thermomixer. Thereafter, the suspension was filtered through a 40µm cell strainer and pelleted by centrifugation at 400g for 5 minutes at 4°C in hank’s balanced salt solution (HBSS) supplemented with 2% FBS (Gibco, 10082-147) and 0.2% bovine serum albumin (PanReac AppliChem, A1391.0100). Red blood cell lysis was performed in RBC Lysis buffer (Biolegend, 420302) for 5 minutes and resuspended in FACS buffer (1x PBS, 2% FCS, 2mM EDTA). Thereafter, cells were stained with anti-CD45.2 BV650 (Biolegend, 109835) primary antibody. Cell sorting was performed on a Sony MA900 Cell Sorter, and gating was done on CD45.2 positive cells with the gate set to capture some CD45.2 negative cells.

scRNA seq of FACS sorted immune cells

Isolated cells from control, cre/+, and Linvemastat treated mice (n=3 each) were sent to the Biomedical Sequencing Facility at the CeMM Research Center for Molecular Medicine of the Austrian Academy of Sciences, who performed library preparation using the Smart-Seq protocol. Sequencing of the prepared libraries was done using the Illumina HiSeq 3000 platform. Raw data, including cell barcode filtering, comparison, and UMI counting were preprocessed and demultiplexed using Cell Ranger (v 7.0.0).

scRNA seq Analysis

Further analysis was conducted with the R package *Seurat* v5.1.0 and was based primarily on the official tutorial. Raw data files were individually loaded, and separate Seurat objects were created. We excluded cells with less than 200 detected genes or with mitochondrial gene content > 20%, and genes presented in less than 10 cells. The data were normalized using the *NormalizeData* function in R. The *FindVariableFeatures* function was used to identify common features between cells, and then the normalized counts were scaled by default. Based on the elbow plot, we performed primary dimensionality reduction using Principal Component Analysis (PCA) with 30 dimensions. Twenty-one clusters were identified using the *FindNeighbors* (based on KNN graphs) and *FindClusters* (based on Louvain method) functions in Seurat. Finally, we used the *RunUMAP* function to perform uniform manifold approximation and projection (UMAP) dimensionality reduction. We used the R package *DoubletFinder* to detect and exclude doublets. The double linkage rate was estimated from the 10X Genomics user guide. Afterwards, the individual Seurat objects were merged, and sample IDs were added to the metadata. Furthermore, we separated cells based on cell cycle scoring using the Ensembl data base to filter out clusters representing different cell states. We used *SCTransform* to remove any batch effects due to differing sequencing depths. Finally, the merged dataset was integrated using the *FindIntegrationAnchors* function in R using the transformed data as a normalization method. The final dimensionality reduction using UMAP was done with a resolution of 0.21 for optimal cluster separation.

The differential expression levels of genes were calculated using *FindMarkers*, which is based on the Wilcoxon ranking and test with default parameters. Clusters were manually annotated using a cell type reference list of gene names. We performed pseudobulk processing for the clusters by aggregating the expression values for each sample and performed Differential gene expression (DEG) analysis using the *DEseq2* function. Functional Gene Set Enrichment (FGSEA) was done using the *fgsea* package in R as indicated in the package vignette. This package enables fast and accurate calculation arbitrarily low gene set enrichment p-values for a collection of gene sets, based on an adaptive multi-level split Monte-Carlo scheme. This was used to identify overrepresented pathways in the data set. Single cell trajectory analysis and pseudotime analysis was done using the *Monocle3* package in R following the official tutorial. To infer possible cell-cell interactions between individual clusters, we used *NicheNet* package in R to model gene expression changes in target cells based on expressed ligand from a sender cell. The package uses prior knowledge of signaling and gene regulatory network based on the expression of certain ligands in the sender cell.

Reanalysis of previously published data sets

We used publicly available scRNA sequencing data sets from Broad Institute Single Cell Portal, including human liver fibrosis atlas (ID SCP2154), human lung fibrosis atlas (ID SCP2155), and mouse liver fibrosis sequencing data (ID SCP2053). Samples from patients with alcoholic liver cirrhosis, hepatocellular carcinoma, non-alcoholic steatosis, primary biliary cholangitis, and control samples. The integrated lung data set contains samples from chronic obstructive pulmonary diseases (COPD), pulmonary fibrosis, pulmonary systemic sclerosis, and controls. The mouse liver fibrosis data set is composed of mouse samples from 2 different fibrosis models, Carbon tetrachloride (CCl4) and High fat diet (HFD) induced liver fibrosis, and samples from chow fed control mice.

In short, raw gene expression matrices were processed using the Seurat package (v4.0). Counts were loaded using the *Read10x* function, and Seurat objects were created using the *CreateSeuratObject* function. Cell metadata was integrated using the *AddMetaData* function in Seurat. We performed quality control by filtering out cells with more than 5% mitochondrial genes and cells expressing less than 200 genes, using the *PercentageFeatueSet* function with the prefix “^MT- “. Myeloid cells were subset based on annotated metadata (Cell_Type== ”Myeloid”). Data were normalized using the LogNormalize method (*NormalizeData*), and the top 2000 variable genes were identified with the variance-stabilizing transformation (*FindVariableFeatures*). Expression values were scaled using the *ScaleData* function.

Dimensionality reduction was done via PCA analysis using the *RunPCA* function, and the number of significant principal components was determined based on elbow plot values. UMAP was conducted on the top 10 principal components by using the *RunUMAP* function. Clustering of cells was done using the shared nearest neighbor (SNN) approach (*FindNeighbors*) and the Louvain algorithm (*FindClusters*). UMAP visualizations of gene expression were done using the *DimPlot* function.

Statistical analysis

Statistical significance was calculated using an unpaired t-test in GraphPad Prism v9.0, with a significance cut off value of p=0.05 for comparisons between two groups. For comparisons between multiple groups (*in vitro* stimulation, ELISA) a one-way analysis of variance (ANOVA), followed by Tukey’s, Šidak’s, or Dunnett’s multiple comparison test were used. Grouped data from *in vitro* MMP12^-/-^ and WT BMDMs were analyzed using ordinary two-way ANOVA with Fisher’s LSD test. Significance: *p<0.05, **p<0.01, ***p<0.001, ****p<0.0001. Quantification of gene expression comparison between two groups from scRNAseq data was done using a Wilcoxon rank sum test. Quantification of DBSCAN cluster sizes of macrophages between two groups was analyzed using a Wilcoxon rank sum test.

Study approval

Ethical approval for using human samples in this study was granted by the London Stanmore National Health Service Research Ethics Committee (reference: 17/LO/0747). Informed consent was provided by next-of-kin at the time of autopsy. All animal experiments have been approved by the official Austrian ethics board (GZ 2023-0.062.018 and GZ 2024-0.896.126).

Data availability

The RNA-sequencing data from hearts of sarcoidosis mice were submitted to the National Center for Biotechnology Information’s Gene Expression Omnibus under accession number GSE292393. No custom code was generated for this study; standard code was used as described in Materials and Methods. Values for all data points in graphs are reported in the Supporting Data Values file. Murine data underlying Figure S6 D & E are openly available in Gene Expression Omnibus under the accession code GSE228232. Human data underlying Fig. 7 E & F are publicly available in the single cell portal of the Broad Institute under the accession codes SCP2154 and SCP2155.

**SUPPLEMENTAL TABLES**

**Supplementary Table 1**. List of top conserved marker genes in cluster 4 in +/+ mice

| Gene | log2FC | % expressing  (cluster 4) | % of expressing  (all other clusters) |
| --- | --- | --- | --- |
| Ccl8 | 4.130842 | 21 | 1.8 |
| Cbr2 | 4.12744 | 44.6 | 4.3 |
| Pla2g2d | 4.010126 | 19.6 | 2 |
| Chp2 | 3.908891 | 16.3 | 2.1 |
| Cdkn2a | 3.791788 | 4.2 | 0.6 |
| Folr2 | 3.449408 | 42.2 | 5.4 |
| Ccl12 | 3.387246 | 18.1 | 2.6 |
| Fcna | 3.374204 | 8.5 | 1.5 |
| Aif1 | 3.350383 | 62.5 | 10.2 |
| Fxyd2 | 3.315405 | 20.5 | 3.5 |
| Ltc4s | 3.222681 | 23 | 3.6 |
| Cela1 | 3.163477 | 5.6 | 1.2 |
| Ms4a7 | 3.150541 | 34.2 | 5.6 |
| C1qc | 3.027565 | 83 | 12.6 |
| Dppa3 | 2.985614 | 1.1 | 0.2 |
| Ccl24 | 2.984363 | 28.6 | 5.1 |
| Prxl2b | 2.971998 | 15 | 4.3 |
| Cd163 | 2.963627 | 27.5 | 4.9 |
| C1qb | 2.924665 | 90.6 | 16.3 |
| C1qa | 2.919809 | 92.2 | 15.9 |

**Supplementary Table 2**. List of top conserved marker genes in cluster 4 in cre/+ mice

| Gene | log2FC | % expressing  (cluster 4) | % expressing  (all other clusters) |
| --- | --- | --- | --- |
| Mmp12 | 6.246134 | 10.5 | 0.3 |
| Cela1 | 5.623667 | 45 | 2.2 |
| Spp1 | 5.318185 | 44.4 | 2.6 |
| Gpnmb | 5.174226 | 54.8 | 3.8 |
| Fxyd2 | 5.071485 | 22.9 | 1.3 |
| Ctsk | 5.007463 | 19.6 | 1.7 |
| Ms4a7 | 4.99422 | 48.9 | 3.2 |
| Speg | 4.86211 | 7.8 | 0.5 |
| Ccl8 | 4.707955 | 23 | 1.7 |
| Barx2 | 4.641387 | 1.2 | 0.1 |
| Ccdc152 | 4.541945 | 18.9 | 1.8 |
| Maoa | 4.47785 | 15 | 1.5 |
| S100a1 | 4.464104 | 72.4 | 9.2 |
| Hebp1 | 4.404885 | 9.9 | 1.1 |
| C1qc | 4.223886 | 51.8 | 4 |
| C1qb | 4.191259 | 60.7 | 5.2 |
| Folr2 | 4.186177 | 18.3 | 1.4 |
| Trem2 | 4.1338 | 47.7 | 6.7 |
| Tceal8 | 4.043815 | 22.2 | 3.2 |
| Flt-ps1 | 3.995599 | 41.6 | 6.3 |

**Supplementary Table 3***.* Experimental design of Everolimus treatment

| # of Mice | Treatment | Dose  (mg/kg) | Mean Age  (weeks) | | Duration  (weeks) |
| --- | --- | --- | --- | --- | --- |
| 4 | Vehicle | *NA* | 41.7 | 3^A^ | |
| 4 | Everolimus | 5 | 41.7 | 3^A^ | |

*NA* Not applicable. ^A^oral gavage once daily.

**Supplementary Table 4*.*** Experimental design of Linvemastat study

| Batch | no. of mice | Treatment | Dose  (mg/kg) | Mean Age  (weeks) | Duration  (weeks) |
| --- | --- | --- | --- | --- | --- |
| 1 | 2 | Vehicle | *NA* | 26 | 3.1^A^ |
| 1 | 12 | Linvemastat | 30 | 26.6 | 3.1^A^ |
| 2 | 6 | Vehicle | *NA* | 62.8 | 3.2^A^ |
| 2 | 5 | Linvemastat | 30 | 56.2 | 3.2^A^ |
| 3 | 7 | Vehicle | *NA* | 39.7 | 3.3^A^ |
| 3 | 7 | Linvemastat | 30 | 40.1 | 3.3^A^ |

^A^via oral gavage twice daily. *NA* Not Applicable.

**Supplementary Table 5***.* Experimental design of Aderamastat treatment

| no. of mice | Treatment | Dose  (mg/kg) | Mean Age  (weeks) | Duration  (weeks) |
| --- | --- | --- | --- | --- |
| 10 | Vehicle | NA | 35.8 | 5^A^ |
| 10 | Aderamastat | 100 | 35.5 | 5^A^ |

^A^via oral gavage twice daily. *NA* Not Applicable.

**Supplementary Table 6*.*** List of animals used for ECG measurements

| no. of mice | Treatment | Dose  (mg/kg) | Mean Age  (weeks) | Duration  (weeks) | Genotype |
| --- | --- | --- | --- | --- | --- |
| 5 | *NA* | *NA* | 42.2 | *NA* | +/+ |
| 6 | Vehicle | *NA* | 41.3 | 5^A^ | cre/+ |
| 5 | Aderamastat | 200 | 39.4 | 5^A^ | cre/+ |

*NA* Not applicable. ^A^oral gavage once daily.

**Supplementary Table 7.** Clinical data of human participants

| Age at Death (years) | Sex | Co-existing conditions | Diagnosis | Pathological Cause of Death |
| --- | --- | --- | --- | --- |
| 33 | Male | None | Sarcoidosis | Sarcoidosis |
| 48 | Female | Asthma | Sarcoidosis | Sarcoidosis |
| 33 | Male | Tuberculosis | Sarcoidosis | Sarcoidosis |
| 41 | Male | Fibromyalgia | Sarcoidosis | Sarcoidosis |
| 35 | Male | None | Sarcoidosis | Sarcoidosis |
| 47 | Female | None | Sarcoidosis | Sarcoidosis |
| 35 | Male | None | Healthy | Suicide |
| 30 | Female | None | Healthy | Trauma |

Figure S1. **Overview of structural changes in the heart**. **A** H&E staining of granulomatous inflammation, marked regions indicate granulomas 200x magnification; **B & C** Immunofluorescence staining and quantification of Mac2 and pS6 of left ventricle, confirming mTORC1 hyperactivation (+/+ n=5, mean age 37.7 weeks, cre/+ n=5, mean age 38.5 weeks) t-test, *** p<0.001, **** p<0.0001; **D** Picrosirius Red (PSR) staining of right atrium showing diffuse fibrosis across the myocardium; **E & F** Immunofluorescence staining and quantification of Collagen 1 staining. The fibrous layer of the atrial and ventricular epicardium is significantly thickened, (+/+ n= 3-4, cre/+ n=6-7, mean age 37.3 weeks), t-test, * p<0.05 ,**** p<0.0001; **G** FAPα immunofluorescence staining of activated fibroblasts, 200x magnification; **H - J** Quantification of fibrosis markers: FAPα, αSMA, and Collagen 1 immunofluorescence staining, (+/+ n=5, cre/+ n=3-7, mean age 38.1 weeks); t-test, * p<0.05, ** p<0.01, *** p<0.001;

Figure S2. **Sequencing of the whole mouse heart reveals major differences in immune cell populations**. **A** Dotplot of major cell types identified. T cells were delineated by *Cd3e*, and with lineage markers such as *Cd4, Cd8a, Il17a*. B cells were stratified using *Cd19, Ccr7, Irf4,* and MHC genes. We used the well-recognized myeloid lineage markers *Lyz2, Csf1r, Itgax, Mmp9, Spp1*, and *Lgals3* to classify macrophages, monocytes, and dendritic cells. Fibroblasts, endothelial cell, platelets, and mast cells were identified based on their highly specific and well described markers such as, *Col1a1*, *Emcn*, *Pf4* (platelet factor 4), and *Kit*, respectively; **B** UMAPs of all samples used for scRNAseq. Green circles indicate the fibrogenic part of cluster 4 found only in the cre/+; **C** Violin plots of key fibrogenic marker genes across all clusters; **D** Enrichment plots of upregulated processes in the FibMacs**; E** Immunofluorescence staining of FibMacs using CD206 and CD163 as a canonical marker of anti-inflammatory macrophage polarization, 400x magnification; **F** Heatmap (cluster 4) of canonical anti-inflammatory markers, proinflammatory genes, and non-classical macrophage markers.

Figure S3: **The spatial and transcriptional landscape of macrophage subpopulations is drastically changed**. **A** Schematic view of isolating the three macrophage and one monocyte cluster; **B** UMAPs of individual samples. FibMacs only arise in cre/+ mice as indicated by the green circles; **C** Cluster 0 in the isolated macrophage clusters is highly enriched for the FibMac genes; **D** Violin plot of *Ccr2* expression between wild type and knock-out mice. cre/+ mice have a significantly higher expression of *Ccr2* compared to wild type controls, Wilcoxon rank-sum test, **** p<0.0001; **E** Heatmap of calculated ligand/gene interactions via *NicheNet* package. It predicts which ligands from a “sender” cell population (Monocytes) influence the expression of target genes in a “receiver” cell type (FibMacs).

Figure S4**. FibMac differentiation from monocytes is regulated by four distinct gene modules**. **A** Heatmap of all 65 Gene expression modules (GEMs), Module 19 contains all fibrogenic markers as well as matrix metalloproteinase and cathepsins signatures; **B** Pseudotemporal gene expression of key transcription factors involved in monocyte-to-macrophage transition*.* Each dot represents one cell. **C** Pseudotemporal gene expression of FibMac marker genes, *Bex1, Cela1,* and *Ctsk.*

Figure S5**. FibMac differentiation from monocytes is regulated by 4 distinct gene modules**. **A - D** Gene Ontology (GO) analysis of all four pseudotime gene modules driving FibMac; **E - H** Heatmaps of genes and associated processes in FibMac differentiation.

Figure S6. **MMP12 expression is highly specific for FibMacs**. **A** Quantification of CD68 and MMP12 expressing macrophages (+/+ n=6, cre/+ n=6), t-test, ** p<0.001, **** p<0.0001; **B** Verhoef van-Gieson (VVG) staining of pulmonary artery cross section, stars indicate granulomatous aggregates, 100x magnification. Lower panel highlights elastin destruction and fragmentation (black arrows) around granulomas (*), 400x magnification; PA – Pulmonary Artery; **C** UMAPs from isolated macrophage clusters, showing high expression of all components of the Elastin Receptor Complex (ERC) specifically in cluster 0 (FibMacs); **D** UMAPs of expression of key fibrogenic genes, along with *Mmp12*, in carbon tetrachloride (CCl4) and high-fat diet models of murine liver fibrosis. FibMac gene activity can be found in mouse models of liver fibrosis; Stars indicate FibMac clusters. **E** Violin plots of *Mmp12, Spp1*, and *Gpnmb* expression between normal and fibrotic liver disease mice. FibMacs (cluster 3) have a significantly higher expression of *Mmp12* compared to wild type controls, Wilcoxon rank-sum test, ** p<0.01, **** p<0.0001.

Figure S7. **MMP12 inhibition aleviates fibrotic burden in the heart**. **A** Design of *in vivo* experiments. The number of mice in each treatment is provided in supplementary tables 3 and 4. Mice were treated once daily with Linvemastat via oral gavage (30 mg/kg) for 3 weeks. Animals treated with Aderamastat received 100mg/kg of the drug twice daily via oral gavage for 5 weeks; **B-E** Sirius Red/Fast Green staining of myocardium and perivascular fibrosis and quantification of fibrotic burden after Linvemastat or Aderamastat and representative Sirius Red – Fast Green stainings, 400x magnification. t- test, *** p<0.001 ,**** p<0.0001; **F & G** Quantification of αSMA and FAPα positive myofibroblasts. Linvemastat treatment alleviated fibrotic burden as well as the numbers of activated myofibroblasts; **H** FGSEA results of upregulated gene sets in FibMacs after Linvemastat treatment. FGSEA analysis was performed on genes ranked by logFC derived from pseudobulk DEG analysis. Gene sets containing 15-500 genes were included. P-values were adjusted using the Benjamini-Hochberg false discovery rate (FDR) method, and pathways with FDR<0.05 were considered significant. The top 15 pathways ranked by normalized enrichment score are shown; **I & J** Quantification and representative images of BMDMs (n=4, mean age 10.5 weeks) stimulated with recombinant MMP12. Classical granuloma markers such as E-Cadherin γ-Catenin were evaluated, t test, ,**** p<0.0001; **K & L** Quantification and representative images of clustering inhibition by Linvemastat (50 µg/mL) in cre/+ BMDMs cultured *in vitro*, (n=4, mean age 10.5 weeks), t-test, * p<0.05.
