# Supplementary material for: MMP12 induces fibrogenic macrophage differentiation to drive granuloma-associated cardiac fibrosis
Source: Am J Respir Crit Care Med. 2026 Jul 29;212(10):2409–23. doi: 10.1093/ajrccm/aamag354 (PMC13624811; doi:10.1093/ajrccm/aamag354)
Supplement: Supplementary file 1 [file aamag354_supplementary_data.zip › Supp_Figures.pdf]

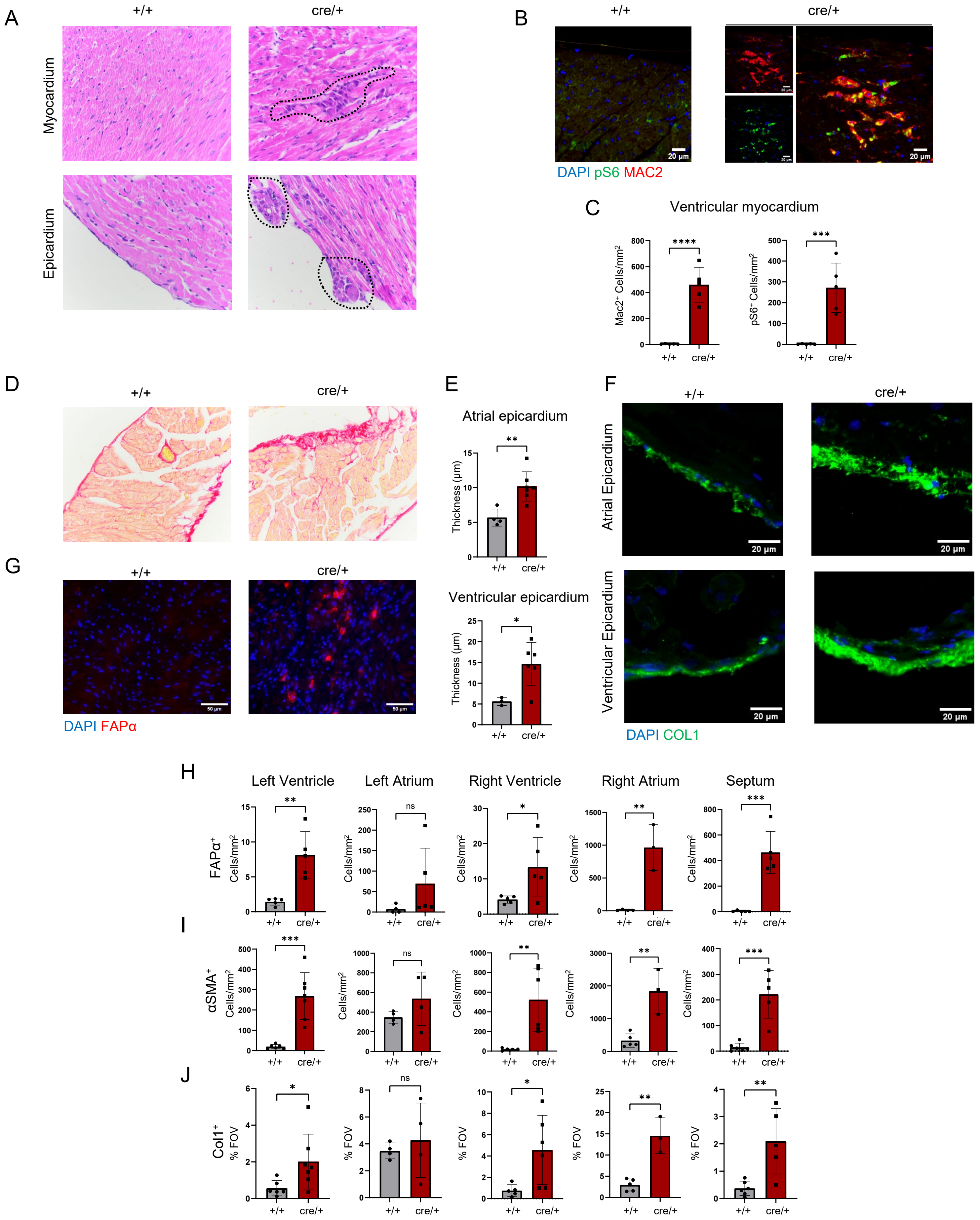

A
+/+
cre/+
Myocardium
Epicardium
B
+/+
cre/+
20 µm
DAPI pS6 MAC2
C
Ventricular myocardium
Mac2+ Cells/mm2
pS6+ Cells/mm2
****
***
+/+
cre/+
D
+/+
cre/+
E
Atrial epicardium
Thickness (µm)
**
Ventricular epicardium
*
F
Atrial Epicardium
Ventricular Epicardium
20 µm
DAPI COL1
G
+/+
cre/+
50 µm
DAPI FAPα
H
Left Ventricle
Left Atrium
Right Ventricle
Right Atrium
Septum
FAPα+
Cells/mm2
ns
I
αSMA+
J
Col1+
% FOV

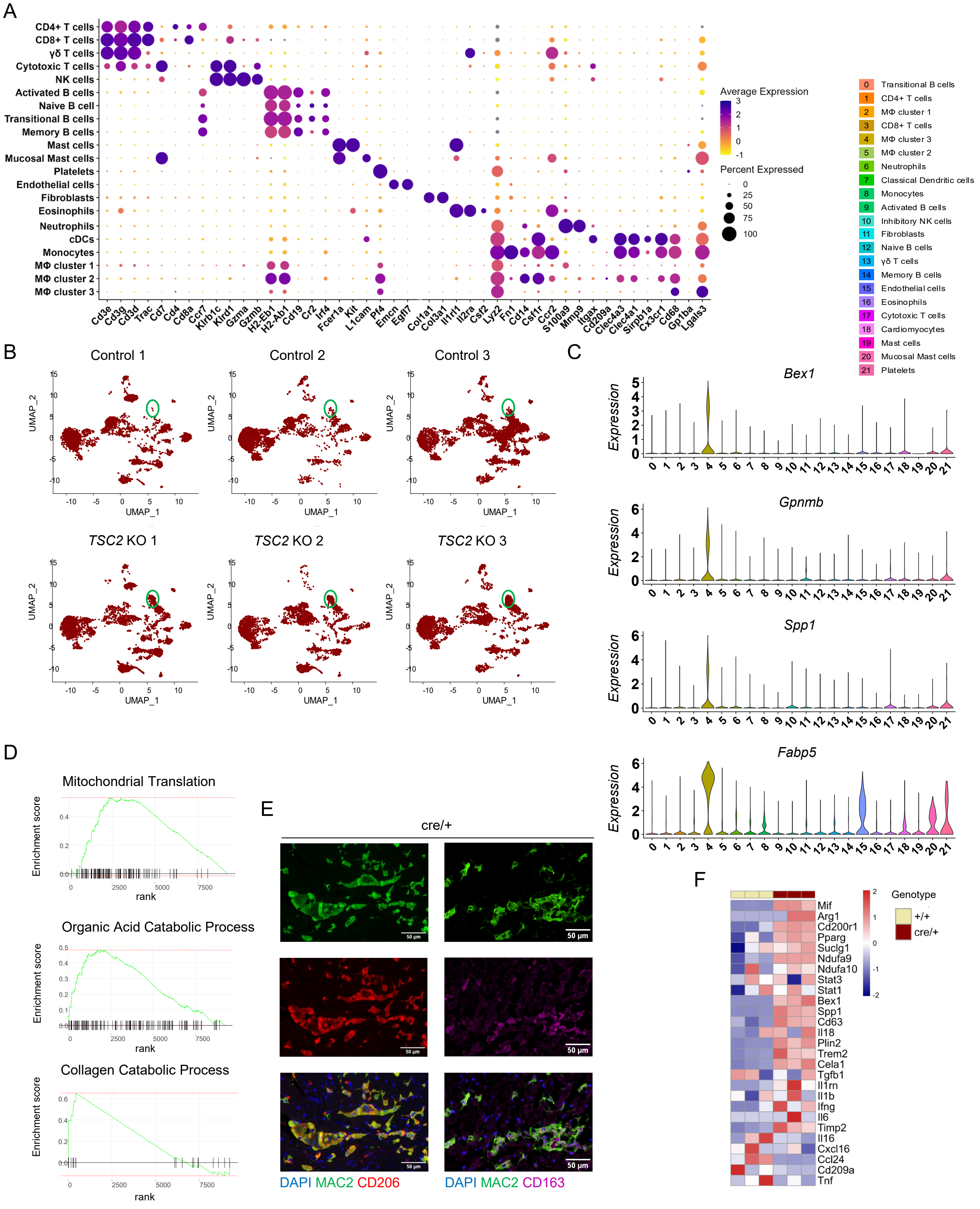
A
CD4+ T cells
CD8+ T cells
γδ T cells
Cytotoxic T cells
NK cells
Activated B cells
Naive B cell
Transitional B cells
Memory B cells
Mast cells
Mucosal Mast cells
Platelets
Endothelial cells
Fibroblasts
Eosinophils
Neutrophils
cDCs
Monocytes
MΦ cluster 1
MΦ cluster 2
MΦ cluster 3
Cd3e Cd3g Cd3d Trac Cd7 Cd4 Cd8a Ccr7 Klrb1c Klrd1 Gzma Gzmb H2-Eb1 H2-Ab1 Cd19 Cr2 Irf4 Fcer1a Kit L1cam Pf4 Emcn Egfl7 Col1a1 Col3a1 Il1rl1 Il2ra Csf2 Lyz2 Fn1 Cd14 Csf1r Ccr2 S100a9 Mmp9 Itgax Cd209a Clec4a3 Clec4a1 Sirpb1a Cx3cr1 Cd68 Gp1ba Lgals3
Average Expression
3
2
1
0
-1
Percent Expressed
0
25
50
75
100
0 Transitional B cells
1 CD4+ T cells
2 MΦ cluster 1
3 CD8+ T cells
4 MΦ cluster 3
5 MΦ cluster 2
6 Neutrophils
7 Classical Dendritic cells
8 Monocytes
9 Activated B cells
10 Inhibitory NK cells
11 Fibroblasts
12 Naive B cells
13 γδ T cells
14 Memory B cells
15 Endothelial cells
16 Eosinophils
17 Cytotoxic T cells
18 Cardiomyocytes
19 Mast cells
20 Mucosal Mast cells
21 Platelets
B
Control 1
Control 2
Control 3
TSC2 KO 1
TSC2 KO 2
TSC2 KO 3
UMAP_1
UMAP_2
C
Bex1
Gpnmb
Spp1
Fabp5
Expression
D
Mitochondrial Translation
Organic Acid Catabolic Process
Collagen Catabolic Process
Enrichment score
rank
E
cre/+
50 μm
DAPI MAC2 CD206
DAPI MAC2 CD163
F
Genotype
+/+
cre/+
Mif
Arg1
Cd200r1
Pparg
Suclg1
Ndufa9
Ndufa10
Stat3
Stat1
Bex1
Spp1
Cd63
Il18
Plin2
Trem2
Cela1
Tgfb1
Il1rn
Il1b
Ifng
Il6
Timp2
Il16
Cxcl16
Ccl24
Cd209a
Tnf

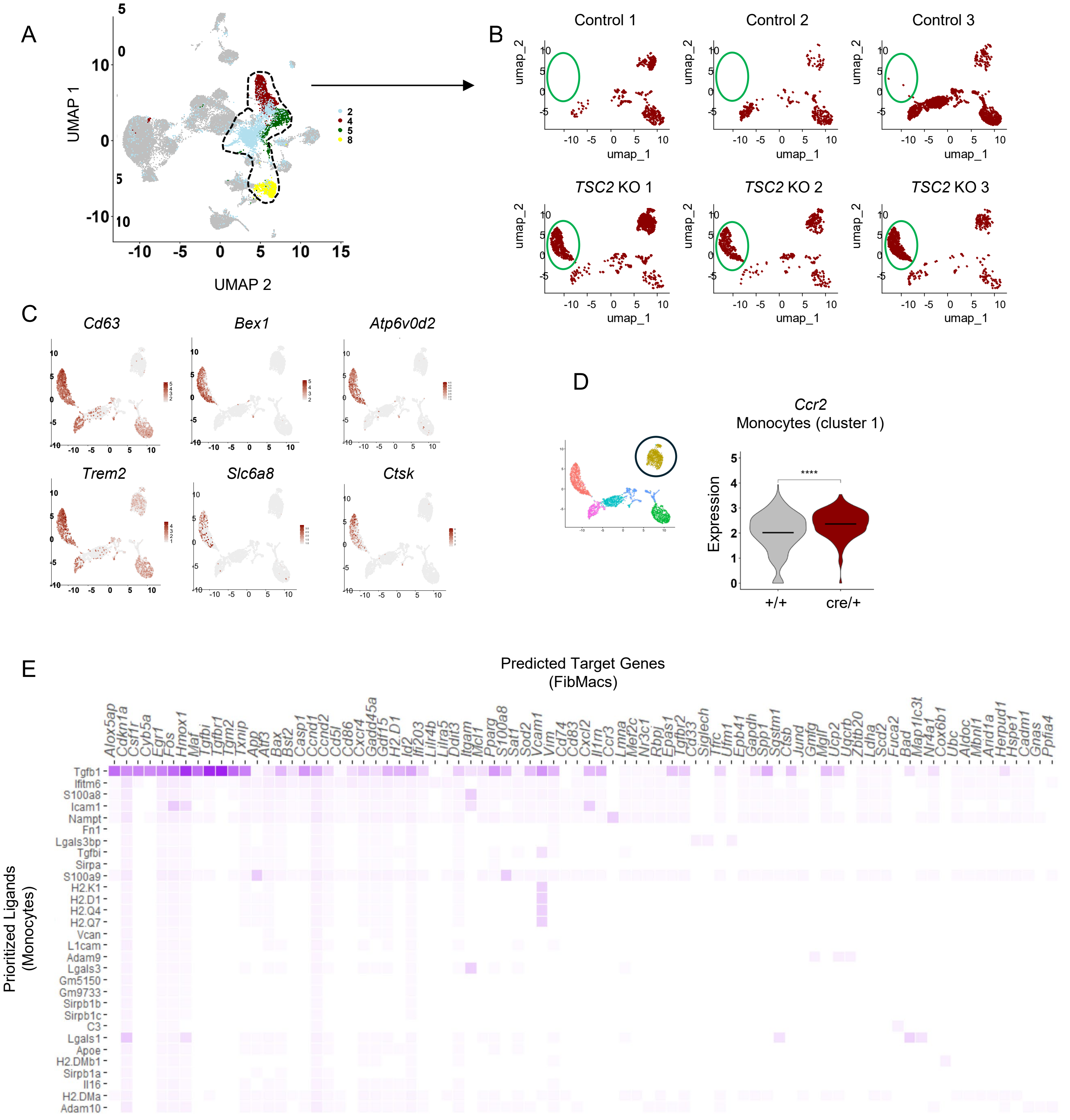

A
UMAP 1
UMAP 2
B
Control 1
Control 2
Control 3
TSC2 KO 1
TSC2 KO 2
TSC2 KO 3
umap_1
umap_2
C
Cd63
Bex1
Atp6v0d2
Trem2
Slc6a8
Ctsk
D
Ccr2
Monocytes (cluster 1)
Expression
+/+
cre/+
E
Predicted Target Genes
(FibMacs)
Prioritized Ligands
(Monocytes)

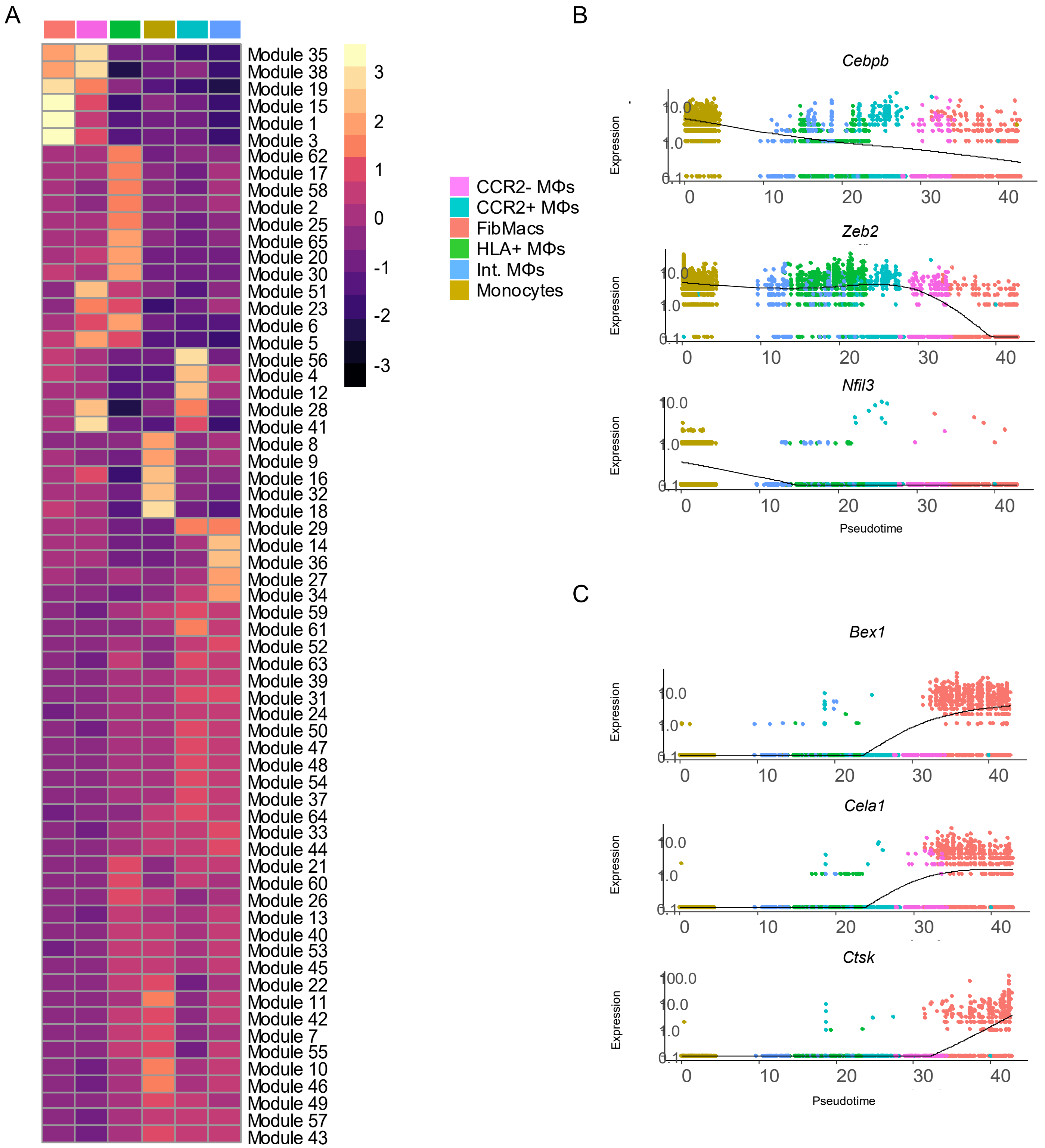

A
B
C
Module 35
Module 38
Module 19
Module 15
Module 1
Module 3
Module 62
Module 17
Module 58
Module 2
Module 25
Module 65
Module 20
Module 30
Module 51
Module 23
Module 6
Module 5
Module 56
Module 4
Module 12
Module 28
Module 41
Module 8
Module 9
Module 16
Module 32
Module 18
Module 29
Module 14
Module 36
Module 27
Module 34
Module 59
Module 61
Module 52
Module 63
Module 39
Module 31
Module 24
Module 50
Module 47
Module 48
Module 54
Module 37
Module 64
Module 33
Module 44
Module 21
Module 60
Module 26
Module 13
Module 40
Module 53
Module 45
Module 22
Module 11
Module 42
Module 7
Module 55
Module 10
Module 46
Module 49
Module 57
Module 43
3
2
1
0
-1
-2
-3
CCR2- MΦs
CCR2+ MΦs
FibMacs
HLA+ MΦs
Int. MΦs
Monocytes
Cebpb
Zeb2
Nfil3
Bex1
Cela1
Ctsk
Expression
Pseudotime
100.0
10.0
1.0
0.1
0
10
20
30
40

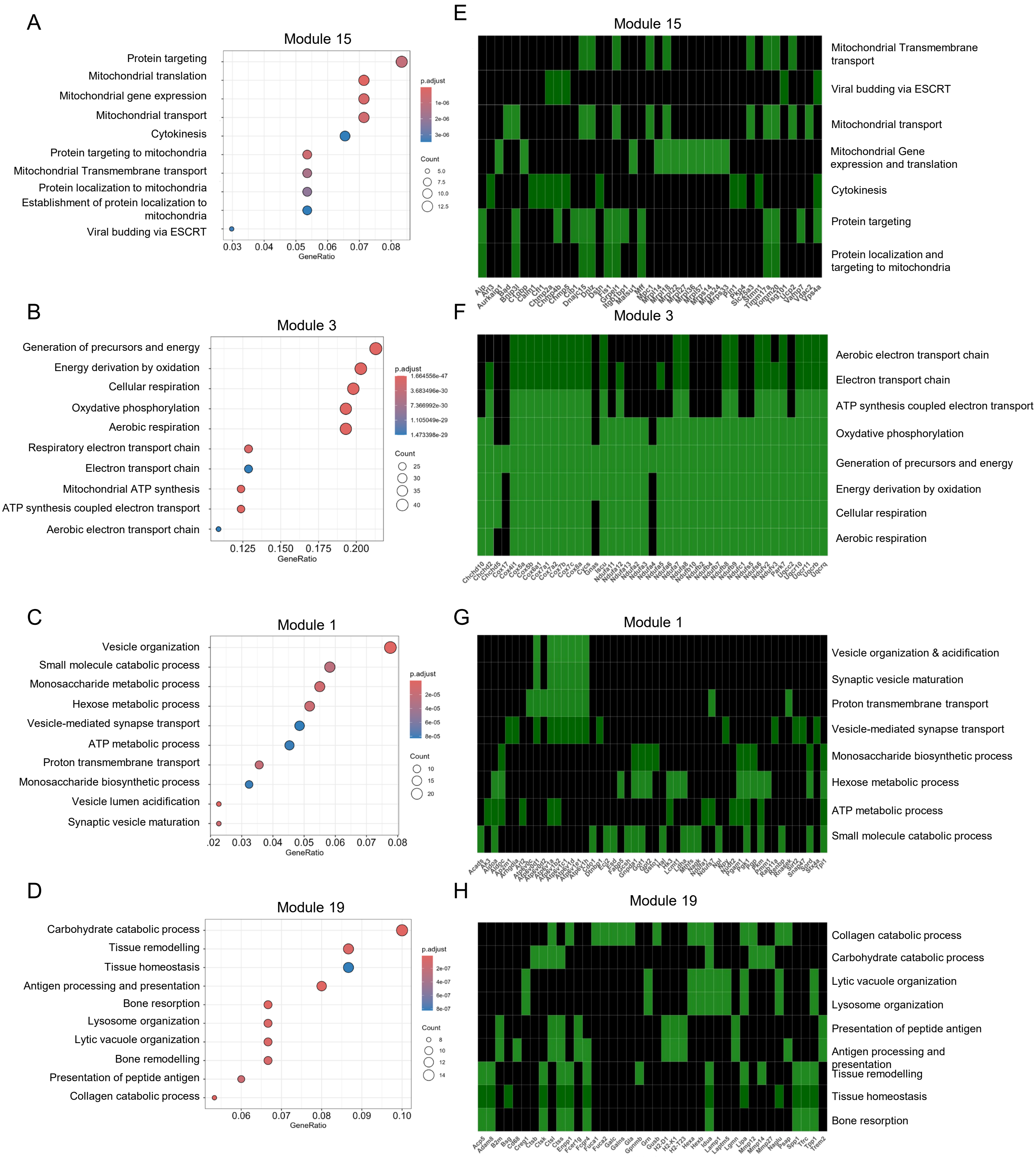

A
Module 15
Protein targeting
Mitochondrial translation
Mitochondrial gene expression
Mitochondrial transport
Cytokinesis
Protein targeting to mitochondria
Mitochondrial Transmembrane transport
Protein localization to mitochondria
Establishment of protein localization to mitochondria
Viral budding via ESCRT
0.03 0.04 0.05 0.06 0.07 0.08
GeneRatio
p.adjust
1e-06
2e-06
3e-06
Count
5.0
7.5
10.0
12.5
E
Module 15
Mitochondrial Transmembrane transport
Viral budding via ESCRT
Mitochondrial transport
Mitochondrial Gene expression and translation
Cytokinesis
Protein targeting
Protein localization and targeting to mitochondria
B
Module 3
Generation of precursors and energy
Energy derivation by oxidation
Cellular respiration
Oxydative phosphorylation
Aerobic respiration
Respiratory electron transport chain
Electron transport chain
Mitochondrial ATP synthesis
ATP synthesis coupled electron transport
Aerobic electron transport chain
0.125 0.150 0.175 0.200
GeneRatio
p.adjust
1.664556e-47
3.683496e-30
7.366992e-30
1.105049e-29
1.473398e-29
Count
25
30
35
40
F
Module 3
Aerobic electron transport chain
Electron transport chain
ATP synthesis coupled electron transport
Oxydative phosphorylation
Generation of precursors and energy
Energy derivation by oxidation
Cellular respiration
Aerobic respiration
C
Module 1
Vesicle organization
Small molecule catabolic process
Monosaccharide metabolic process
Hexose metabolic process
Vesicle-mediated synapse transport
ATP metabolic process
Proton transmembrane transport
Monosaccharide biosynthetic process
Vesicle lumen acidification
Synaptic vesicle maturation
.02 0.03 0.04 0.05 0.06 0.07 0.08
GeneRatio
p.adjust
2e-05
4e-05
6e-05
8e-05
Count
10
15
20
G
Module 1
Vesicle organization & acidification
Synaptic vesicle maturation
Proton transmembrane transport
Vesicle-mediated synapse transport
Monosaccharide biosynthetic process
Hexose metabolic process
ATP metabolic process
Small molecule catabolic process
D
Module 19
Carbohydrate catabolic process
Tissue remodelling
Tissue homeostasis
Antigen processing and presentation
Bone resorption
Lysosome organization
Lytic vacuole organization
Bone remodelling
Presentation of peptide antigen
Collagen catabolic process
0.06 0.07 0.08 0.09 0.10
GeneRatio
p.adjust
2e-07
4e-07
6e-07
8e-07
Count
8
10
12
14
H
Module 19
Collagen catabolic process
Carbohydrate catabolic process
Lytic vacuole organization
Lysosome organization
Presentation of peptide antigen
Antigen processing and presentation
Tissue remodelling
Tissue homeostasis
Bone resorption

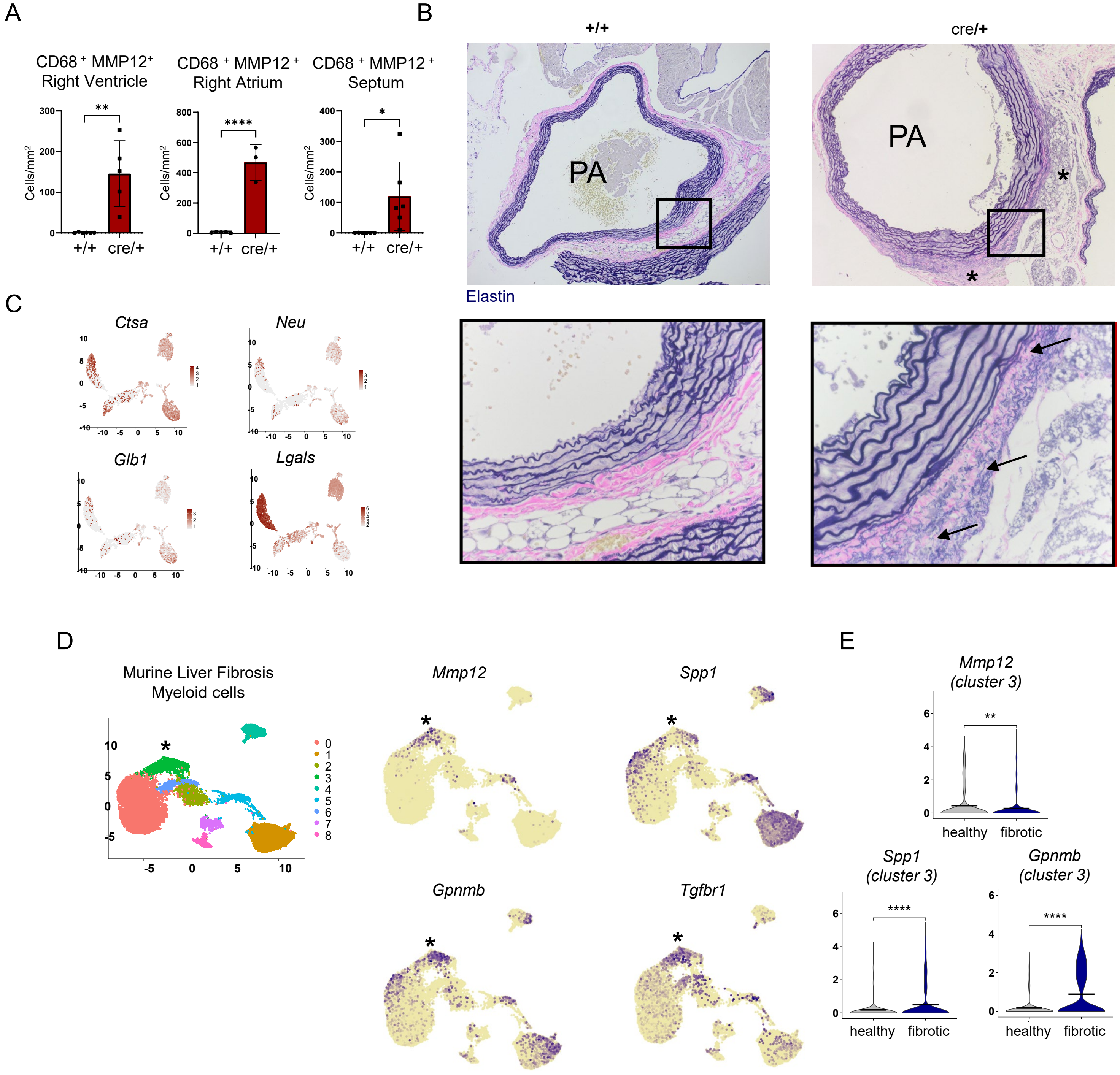
A
CD68 + MMP12+ Right Ventricle
CD68 + MMP12 + Right Atrium
CD68 + MMP12 + Septum
Cells/mm2
+/+
cre/+
B
+/+
cre/+
PA
Elastin
C
Ctsa
Neu
Glb1
Lgals
D
Murine Liver Fibrosis Myeloid cells
Mmp12
Spp1
Gpnmb
Tgfbr1
E
Mmp12 (cluster 3)
Spp1 (cluster 3)
Gpnmb (cluster 3)
healthy
fibrotic

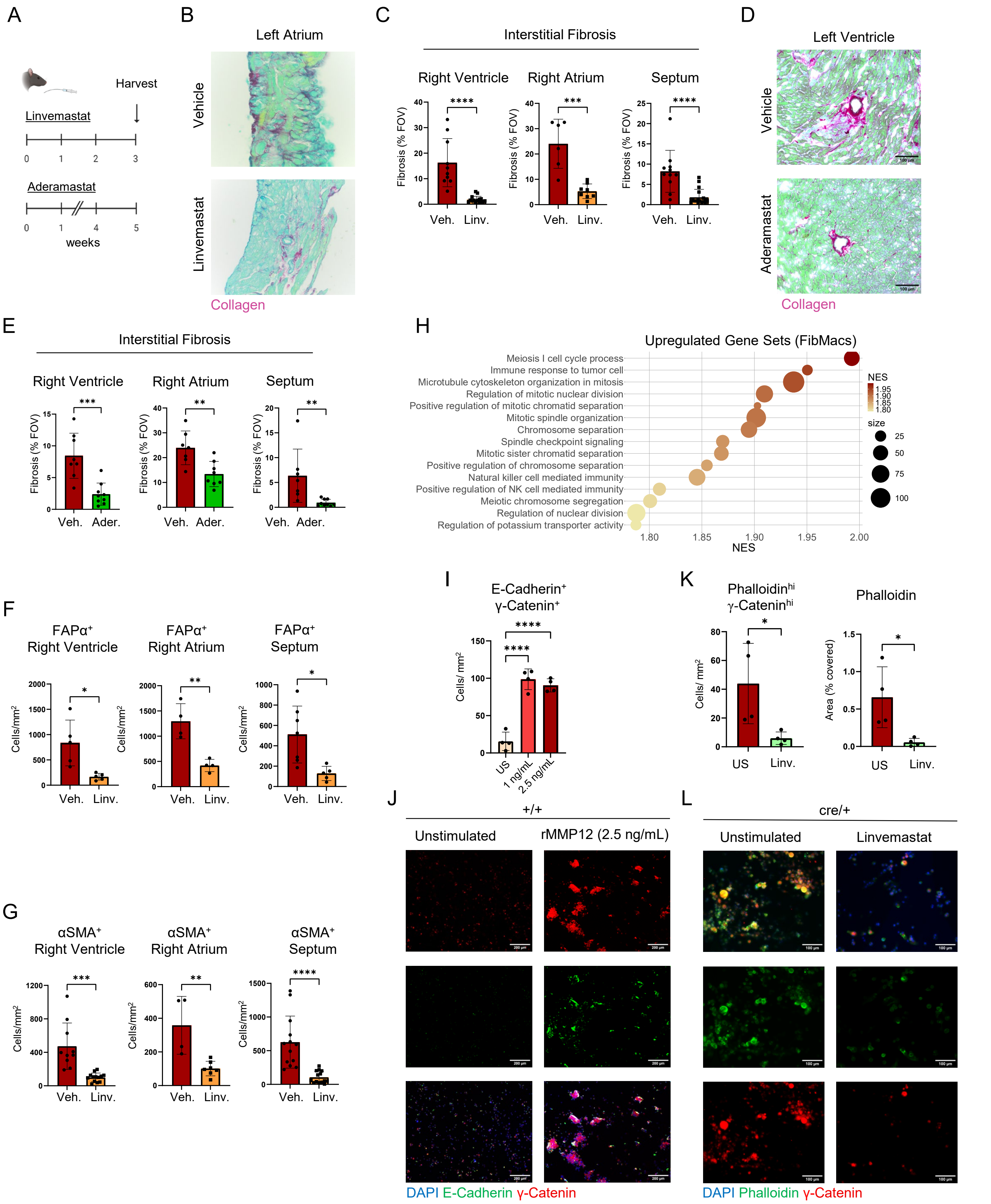

A
Harvest
Linvemastat
0 1 2 3
Aderamastat
0 1 4 5
weeks
B
Left Atrium
Vehicle
Linvemastat
Collagen
C
Interstitial Fibrosis
Right Ventricle
Right Atrium
Septum
Fibrosis (% FOV)
Veh. Linv.
D
Left Ventricle
Vehicle
Aderamastat
Collagen
E
Interstitial Fibrosis
Right Ventricle
Right Atrium
Septum
Fibrosis (% FOV)
Veh. Ader.
H
Upregulated Gene Sets (FibMacs)
Meiosis I cell cycle process
Immune response to tumor cell
Microtubule cytoskeleton organization in mitosis
Regulation of mitotic nuclear division
Positive regulation of mitotic chromatid separation
Mitotic spindle organization
Chromosome separation
Spindle checkpoint signaling
Mitotic sister chromatid separation
Positive regulation of chromosome separation
Natural killer cell mediated immunity
Positive regulation of NK cell mediated immunity
Meiotic chromosome segregation
Regulation of nuclear division
Regulation of potassium transporter activity
NES
size
NES
F
FAPα+ Right Ventricle
FAPα+ Right Atrium
FAPα+ Septum
Cells/mm2
Veh. Linv.
I
E-Cadherin+ γ-Catenin+
Cells/ mm2
US 1 ng/mL 2.5 ng/mL
K
Phalloidinhi γ-Cateninhi
Phalloidin
Cells/ mm2
Area (% covered)
US Linv.
G
αSMA+ Right Ventricle
αSMA+ Right Atrium
αSMA+ Septum
Cells/mm2
Veh. Linv.
J
+/+
Unstimulated
rMMP12 (2.5 ng/mL)
DAPI E-Cadherin γ-Catenin
L
cre/+
Unstimulated
Linvemastat
DAPI Phalloidin γ-Catenin

Gene	log2FC	% expressing (cluster 4)	% of expressing (all other clusters)
Ccl8	4.130842	21	1.8
Cbr2	4.12744	44.6	4.3
Pla2g2d	4.010126	19.6	2
Chp2	3.908891	16.3	2.1
Cdkn2a	3.791788	4.2	0.6
Folr2	3.449408	42.2	5.4
Ccl12	3.387246	18.1	2.6
Fcna	3.374204	8.5	1.5
Aif1	3.350383	62.5	10.2
Fxyd2	3.315405	20.5	3.5
Ltc4s	3.222681	23	3.6
Cela1	3.163477	5.6	1.2
Ms4a7	3.150541	34.2	5.6
C1qc	3.027565	83	12.6
Dppa3	2.985614	1.1	0.2
Ccl24	2.984363	28.6	5.1
Prxl2b	2.971998	15	4.3
Cd163	2.963627	27.5	4.9
C1qb	2.924665	90.6	16.3
C1qa	2.919809	92.2	15.9

Supplementary Table 1. List of top conserved marker genes in cluster 4 in +/+ mice

Gene	log2FC	% expressing (cluster 4)	% of expressing (all other clusters)
Mmp12	6.246134	10.5	0.3
Cela1	5.623667	45	2.2
Spp1	5.318185	44.4	2.6
Gpnmb	5.174226	54.8	3.8
Fxyd2	5.071485	22.9	1.3
Ctsk	5.007463	19.6	1.7
Ms4a7	4.99422	48.9	3.2
Speg	4.86211	7.8	0.5
Ccl8	4.707955	23	1.7
Barx2	4.641387	1.2	0.1
Ccdc152	4.541945	18.9	1.8
Maoa	4.47785	15	1.5
S100a1	4.464104	72.4	9.2
Hebp1	4.404885	9.9	1.1
C1qc	4.223886	51.8	4
C1qb	4.191259	60.7	5.2
Folr2	4.186177	18.3	1.4
Trem2	4.1338	47.7	6.7
Tceal8	4.043815	22.2	3.2
Flt-ps1	3.995599	41.6	6.3

Supplementary Table 2. List of top conserved marker genes in cluster 4 in cre/+ mice

# of Mice	Treatment	Dose (mg/kg)	Mean Age (weeks)	Duration (weeks)
4	Vehicle	*NA*	41.7	3[A]
4	Everolimus	5	41.7	3[A]

NA Not applicable. [A]oral gavage once daily.

Supplementary Table 3. Experimental design of Everolimus treatment

Batch	no. of Mice	Treatment	Dose (mg/kg)	Mean Age (weeks)	Duration (weeks)
1	2	Vehicle	*NA*	26	3.1[A]
1	12	Linvemastat	30	26.6	3.1[A]
2	6	Vehicle	*NA*	62.8	3.2[A]
2	5	Linvemastat	30	56.2	3.2[A]
3	7	Vehicle	*NA*	39.7	3.3[A]
3	7	Linvemastat	30	40.1	3.3[A]

[A]via oral gavage twice daily. *NA* Not Applicable.

Supplementary Table 4. Experimental design of Linvemastat study

no. of Mice	Treatment	Dose (mg/kg)	Mean Age (weeks)	Duration (weeks)
10	Vehicle	NA	35.8	5[A]
10	Aderamastat	100	35.5	5[A]

[A]via oral gavage twice daily. *NA* Not Applicable.

Supplementary Table 5. Experimental design of Aderamastat treatment

# of Mice	Treatment	Dose (mg/kg)	Mean Age (weeks)	Duration (weeks)	Genotype
5	*NA*	*NA*	42,2	*NA*	+/+
6	*NA*	*NA*	42,8	*NA*	Cre/+
6	Vehicle	*NA*	41.3	5[A]	Cre/+
5	Aderamastat	200 mg/kg	39.4	5[A]	Cre/+

NA Not applicable. [A]oral gavage once daily.

Supplementary Table 6. List of animals used for ECG measurements

Age at Death (yr)	Sex	Co-existing conditions	Diagnosis	Pathological Cause of Death
33	Male	None	Sarcoidosis	Sarcoidosis
48	Female	Asthma	Sarcoidosis	Sarcoidosis
33	Male	Tuberculosis	Sarcoidosis	Sarcoidosis
41	Male	Fibromyalgia	Sarcoidosis	Sarcoidosis
35	Male	None	Sarcoidosis	Sarcoidosis
47	Female	None	Sarcoidosis	Sarcoidosis
35	Male	None	Healthy	Suicide
30	Female	None	Healthy	Trauma

Supplementary Table 7. Clinical data of human participants
